# Spatiotemporal integration of looming visual and tactile stimuli near the face

**DOI:** 10.1002/hbm.23995

**Published:** 2018-02-06

**Authors:** Ruey‐Song Huang, Ching‐fu Chen, Martin I. Sereno

**Affiliations:** ^1^ Institute for Neural Computation, University of California, San Diego La Jolla California; ^2^ Department of Electrical and Computer Engineering University of California, San Diego La Jolla California; ^3^ Department of Psychology and Neuroimaging Center San Diego State University San Diego California; ^4^ Experimental Psychology University College London London UK

**Keywords:** approaching threats, binding problem, 4D film, fMRI, neurocinematics, multisensory integration, peripersonal space, psychophysics

## Abstract

Real‐world objects approaching or passing by an observer often generate visual, auditory, and tactile signals with different onsets and durations. Prompt detection and avoidance of an impending threat depend on precise binding of looming signals across modalities. Here we constructed a multisensory apparatus to study the spatiotemporal integration of looming visual and tactile stimuli near the face. In a psychophysical experiment, subjects assessed the subjective synchrony between a looming ball and an air puff delivered to the same side of the face with a varying temporal offset. Multisensory stimuli with similar onset times were perceived as completely out of sync and assessed with the lowest subjective synchrony index (SSI). Across subjects, the SSI peaked at an offset between 800 and 1,000 ms, where the multisensory stimuli were perceived as optimally in sync. In an fMRI experiment, tactile, visual, tactile‐visual out‐of‐sync (TVoS), and tactile‐visual in‐sync (TViS) stimuli were delivered to either side of the face in randomized events. Group‐average statistical responses to different stimuli were compared within each surface‐based region of interest (sROI) outlined on the cortical surface. Most sROIs showed a preference for contralateral stimuli and higher responses to multisensory than unisensory stimuli. In several bilateral sROIs, particularly the human MT+ complex and V6A, responses to spatially aligned multisensory stimuli (TVoS) were further enhanced when the stimuli were in‐sync (TViS), as expressed by TVoS < TViS. This study demonstrates the perceptual and neural mechanisms of multisensory integration near the face, which has potential applications in the development of multisensory entertainment systems and media.

## 1 INTRODUCTION

We perceive the world around us through multiple senses. Concurrent impulses of signals in different sensory modalities, such as a flash and a beep, can readily be merged into a single, static event. However, real‐world dynamic events often generate signals with different onsets and durations across modalities (Spence & Squire, 2003; Vroomen & Keetels, 2010). The optimal binding of cross‐modal signals may take place at anytime during a dynamic event, not just its onset (Bushara et al., 2003). For example, the onset of a continuous siren warns the driver about an approaching ambulance, but its traveling directions can only be definitely confirmed at a later moment when the driver detects its flashing lights. Determining whether multisensory looming signals with different onsets and durations originate from a common source is critical for prompt detection and avoidance of an impending threat (Billington, Wilkie, Field, & Wann, 2011; Burr, Silva, Cicchini, Banks, & Morrone, 2009; De Haan, Smit, Van der Stigchel, & Dijkerman, 2016; De Paepe, Crombez, & Legrain, 2016; Graziano & Cooke, 2006; Holt et al., 2014; Neppi‐Modona, Auclair, Sirigu, & Duhamel, 2004; Poljac, Neggers, & van den Berg, 2006).

Monkey neurophysiological and human neuroimaging studies have revealed multiple cortical areas playing important roles in multisensory integration: (1) middle temporal complex (MT+), including the medial superior temporal area (MST) (Beauchamp, Yasar, Kishan, & Ro, 2007; Blake, Sobel, & James, 2004; Hagen et al., 2002); (2) superior temporal sulcus (STS) (Beauchamp, Argall, Bodurka, Duyn, & Martin, 2004a; Beauchamp, Lee, Argall, & Martin, 2004b; Beauchamp, Yasar, Frye, & Ro, 2008; Calvert, 2001; Calvert, Hansen, Iversen, & Brammer, 2001; Maier, Chandrasekaran, & Ghazanfar, 2008; Marchant, Ruff, & Driver, 2012; Seifritz et al., 2002; Tyll et al., 2013); (3) ventral intraparietal area (VIP) (Avillac, Deneve, Olivier, Pouget, & Duhamel, 2005; Avillac, Ben Hamed, & Duhamel, 2007; Bremmer et al., 2001; Colby, Duhamel, & Goldberg, 1993; Duhamel, Colby, & Goldberg, 1998; Huang, Chen, Tran, Holstein, & Sereno, 2012; Huang, Chen, & Sereno, 2017; Ishida, Nakajima, Inase, & Murata, 2010; McCollum, Klam, & Graf, 2012; Sereno & Huang, 2006); (4) precentral polysensory zone (PZ) and ventral premotor cortex (PMv) (Bremmer et al., 2001; Fogassi et al., 1996; Graziano & Gandhi, 2000; Graziano, Yap, & Gross, 1994; Graziano, Hu, & Gross, 1997; Huang & Sereno, 2007, 2018); and (5) area 7b at the posterior lateral sulcus (Dong, Chudler, Sugiyama, Roberts, & Hayashi, 1994; Graziano, Gross, Taylor, & Moore, 2004; Ishida et al., 2010). Most of these multisensory areas also respond to visual, auditory, and/or tactile motion, including looming stimuli. While the spatial integration of looming visual and auditory stimuli has been demonstrated in previous studies (Cappe, Thut, Romei, & Murray, 2009; Cappe, Thelen, Romei, Thut, & Murray, 2012; Maier et al., 2008; Tyll et al., 2013), few have studied the temporal integration of looming visual and tactile stimuli in near‐body space (Cléry, Guipponi, Odouard, Wardak, & Ben Hamed, 2015).

In this study, we conducted psychophysical and functional magnetic resonance imaging (fMRI) experiments to investigate the temporal integration of spatially aligned looming visual and tactile stimuli near the face. We used wide‐field virtual reality to simulate balls looming toward and passing by the face. Following the onset of a looming ball, an air puff was delivered tangentially to the cheek with a varying temporal offset. In a psychophysical experiment, subjects assessed the subjective synchrony between the looming visual and tactile stimuli in each trial. The optimal temporal offset where the multisensory stimuli were subjectively perceived and interpreted as being originating from the same physical event (i.e., they are in sync with each other) was estimated for each individual subject. About half of the subjects also participated in an fMRI experiment presented with randomized events containing unisensory (tactile only or visual only) or multisensory (tactile and visual; out‐of‐sync or in‐sync) stimuli. Statistical maps of brain activations rendered on cortical surfaces were averaged across subjects using spherical morphing and averaging methods, and surface‐based regions of interest (sROIs) were outlined in the group‐average maps. For each sROI, we compared group‐average statistics between ipsilateral and contralateral stimuli, between tactile and visual stimuli, between unisensory and multisensory stimuli, and between out‐of‐sync and in‐sync multisensory stimuli. While previous studies have demonstrated that spatially aligned multisensory stimuli elicit stronger activations in contralateral brain regions, this study aims to determine whether temporally synchronized multisensory stimuli further enhances the activation.

## 2 MATERIALS AND METHODS

### 2.1 Participants

Twenty healthy right‐hand dominant subjects (19–23 years; 9 males, 11 females) with normal or corrected‐to‐normal vision participated in this study. All subjects participated in a psychophysical session, and 11 of them also participated in an fMRI session on a later day. All subjects gave written informed consent according to protocols approved by the Human Research Protections Program of the University of California, San Diego (UCSD).

### 2.2 Apparatus and stimuli

**Figure 1:**
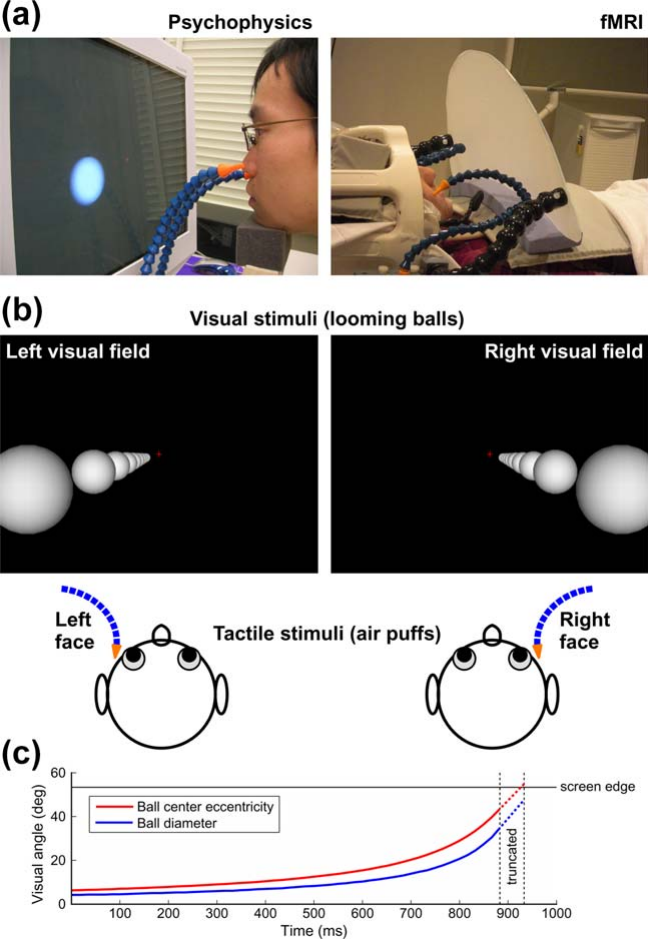
Experimental setup and stimuli. (a) Multisensory stimulation apparatus in psychophysical and fMRI experiments. (b) Spatially aligned looming visual and tactile stimuli near either side of the face. The trace of a looming ball was created by superimposing key frames between 0 and 900 ms, where only one ball was visible per frame (see Supporting Information Figure S1a). (c) Time courses of the eccentricity and diameter of a looming ball. The ball was truncated between 883 and 933 ms (dashed segments) before disappearing at the screen edge [Color figure can be viewed at http://wileyonlinelibrary.com]

We designed and constructed a multisensory apparatus to deliver spatially aligned looming visual and tactile stimuli near the face in psychophysical and fMRI experiments (Figure 1). Visual stimuli (looming balls) were simulated in a virtual reality environment written in the C language using the OpenGL Performer library (Huang et al., 2012; Huang, Chen, & Sereno, 2015; Huang et al., 2017), and rendered at a resolution of 1,024 × 768 pixels with a refresh rate of 60 Hz on a two‐dimensional (2D) CRT monitor (psychophysical experiment) or on an LCD projector (fMRI experiment). Tactile stimuli (100‐ms air puffs; 50–55 psi at air cylinder output) were delivered tangentially to either side of the face via a flexible hose (1/4‐in. inside diameter; Loc‐Line, Lockwood Products Inc., OR) ending with a 1/16‐in. opening (orange nozzles in Figure 1a). The hardware and software for controlling the tactile stimuli were detailed in our previous study (Huang & Sereno, 2007). Both visual and tactile stimuli were controlled by a C language program running on a Linux‐based stimulus computer, and electronic and pneumatic delays between visual and tactile stimuli were precisely calibrated.

### 2.3 Psychophysical experiment

#### 2.3.1 Experimental design and stimuli

Twenty subjects participated in a psychophysical experiment consisting of two calibration runs, one practice run, and four actual runs. The subject placed his or her chin on a chinrest 15 cm in front of a 21‐in. CRT monitor (Sony Multiscan E540; Figure 1a, left), with a visible width of 39‐cm subtending a horizontal field of view of 104.8° (52.4° maximum eccentricity; Figure 1c). At the onset of each trial in all runs, a white ball appeared with equal probability on either side of a fixation cross, with its center located at 15° below the horizon (Figure 1b). Between 0 and 883 ms, the ball's center traveled from 6.3° to 43.5° in eccentricity along a straight path 15° below the horizon, with its diameter expanding from 4.2° to 34.8° (Figure 1b and c; see key frames in Supporting Information Figure S1a). The ball reached the screen edge and then completely disappeared at 933 ms (Figure 1c).

To deliver tactile stimuli, each flexible hose mounted on the base of the chinrest was initially bent toward the subject's cheek with its nozzle pointing tangentially to the skin surface (Figure 1a and b). In repeated trials of the first calibration run, a 100‐ms air puff was delivered to the left cheek at 800 ms following the onset of a looming ball, where it was subjectively perceived and interpreted as wind caused by the ball apparently passing by the left face. The subject fixated a central cross while manually adjusting the left nozzle to align the air puff with the looming ball. These procedures were then repeated for right‐face stimuli in the second calibration run.

**Figure 2:**
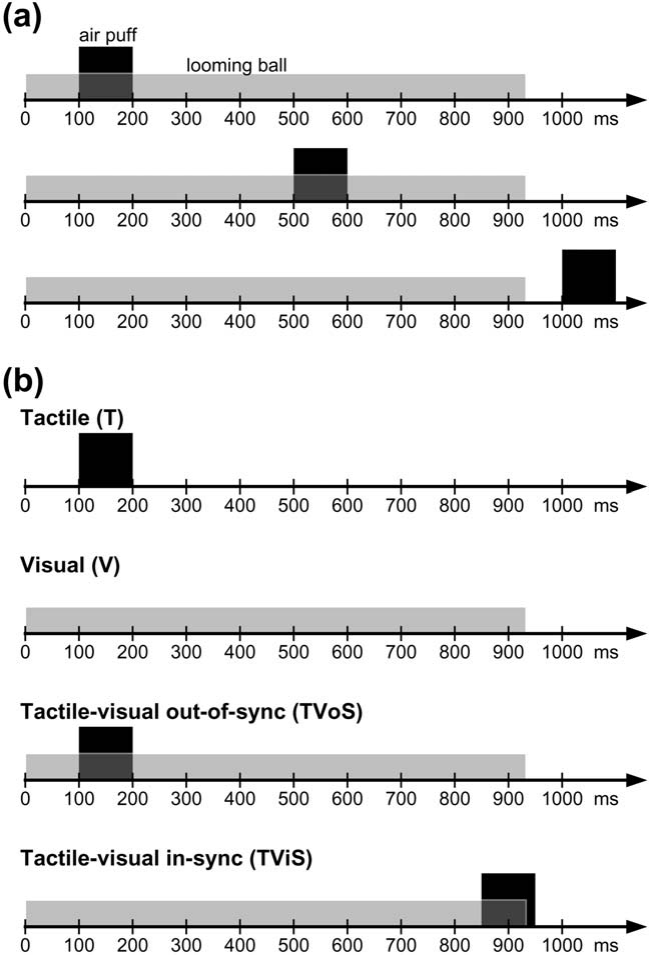
Experimental paradigms and stimulus timelines. (a) Three representative pairs of multisensory stimuli with different temporal offsets (100, 500, and 1,000 ms) in the psychophysical experiment. (b) Unisensory and multisensory stimuli in the fMRI experiment. Black square: duration of an air puff; Gray bar: duration of a looming ball

In the practice run, spatially aligned looming balls and air puffs were delivered in 50 trials with equal probability on each side. In each trial, a 100‐ms air puff was delivered with a temporal offset, randomized between 100 and 1,000 ms (step = 100 ms) following the onset (0 ms) of a looming ball (Figure 2a). Air puffs were not delivered at 0 ms because: (1) in the real world, it takes time for a distant object to approach an observer and cause a tactile impact on the body (direct hit or brushing); (2) it takes time for the visual system to detect and process a moving object; (3) the stimulus processing time is different between visual and somatosensory systems; and (4) improbable events (due to the above reasons) were ruled out to reduce the overall number of trials. The subject then participated in four actual runs (50 trials per run) with the same stimuli as in the practice run, and with a short break between runs. Twenty stimulus conditions (10 offsets × 2 sides × 10 occurrences) were balanced and randomized in 200 trials across four runs.

In each trial of the practice and actual runs, a white scroll bar overlaid with a red cursor appeared above the fixation cross immediately after both stimuli disappeared (Supporting Information Figure S1b). The subject moved the cursor with arrow keys on a standard keyboard to assess the subjective synchrony between each pair of visual and tactile stimuli. The subject was instructed to determine whether both stimuli originate from the same physical event (i.e., a common source), rather than assessing the temporal alignment between stimulus onsets. The subject was also instructed to take time to make the best assessment using the full range of the scroll bar representing a visual analog scale: leftmost = 0, completely out of sync; rightmost = 1, optimally in sync. Once an assessment was made, the subject resumed fixating the central cross and pressed a space bar to proceed to the next trial. The final cursor position on the scroll bar in each trial was automatically recorded in a log file on the same stimulus computer, and then converted into a subjective synchrony index (SSI) between 0 and 1.

#### 2.3.2 Behavioral data analysis

**Figure 3:**
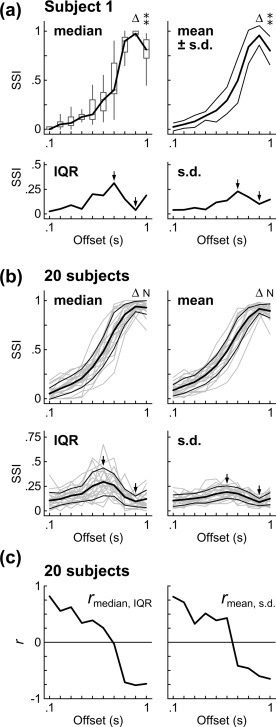
Results of the psychophysical experiment. (a) SSI curves of a representative subject. Upper‐left panel: The bottom and top of each box respectively represent the first and third quartiles of the distribution of 20 SSIs. The height of each box indicates the interquartile range (IQR). Each whisker indicates 1.5 IQR above or below the box. The SSI‐median curve connects the mark (median) within each box. Δ: peak. **: significant decrease from the peak, *p* < .01, Bonferroni corrected. N: insignificant decrease from the peak. (b) SSI curves (thin gray curves) of 20 subjects, overlaid with group mean (a thick black curve) ± standard deviation (thin black curves) in each panel. (c) Correlation coefficients (*r*) between 20 pairs of SSI‐median and SSI‐IQR curves and between 20 pairs of SSI‐mean and SSI‐s.d. curves in (b)

For each subject, 20 trials (left‐face and right‐face stimuli combined) with the same temporal offset were extracted and grouped across four actual runs (practice run not analyzed). The distribution of SSIs in 20 trials at each temporal offset are illustrated by box plots (with median and interquartile range [IQR]), mean, and standard deviation (s.d.), as shown in Figure 3a (Subject 1) and Supporting Information Figures S2 and S3 (Subjects 2–20). Single‐subject SSI median, IQR, mean, and s.d. curves (Figure 3a; Supporting Information Figures S2 and S3) were further averaged across 20 subjects to yield the group trends (group mean ± s.d. curves) for each measure (Figure 3b).

To estimate the temporal offset where the looming visual and tactile stimuli were perceived as optimally in sync by each individual, 100 same‐side (left‐face or right‐face) trials in each subject were grouped and sorted by SSI. The temporal offsets in the top 10 trials (10%) with the highest SSIs were averaged for each side, and the results were further averaged across both sides to yield an estimate of the optimal temporal offset for each subject (Supporting Information Table S1).

#### 2.3.3 Behavioral data modeling

**Figure 4:**
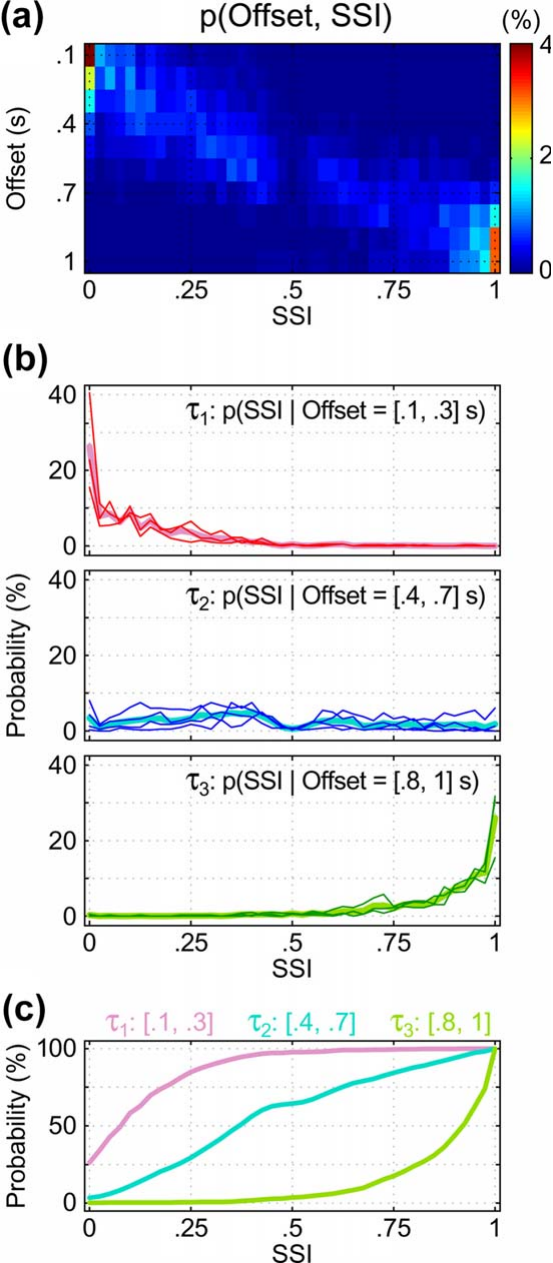
Results of behavioral data modeling. (a) Joint probability of *T* (Offset) and *S* (SSI), p(T, S). The color bar represents the joint probability of each $\left(t_{m},\;s_{n}\right)$ pair. Each row of the 2D plot represents a curve of the joint probability from a bird's eye view. (b) The probability of an SSI, $s_{n}$, conditioned on a temporal offset, $t_{m}$: $p\left(S|T=t_{m}\right)$ (thin curves); within‐group conditional probability: p(S|T∈τ) (thick curves). Each sub‐plot represents a group obtained by applying the *k*‐means algorithm to $p\left(S|T=t_{m}\right)$ curves. (c) Curves of cumulative distribution $F\left(S\leq s_{n}|T\in \tau \right)$ matching the groups τ_1_ to τ_3_ in (b) [Color figure can be viewed at http://wileyonlinelibrary.com]

To investigate the overall distribution of SSIs assessed at each temporal offset, all trials (left‐face and right‐face combined) across 20 subjects were grouped to form 4,000 pairs of (T, S), where $T\in {\left\{t_{m}\right\}}_{m=1}^{10}=\left\{\;t_{1},\ldots ,t_{10}\right\}$ represents temporal offsets between 100 and 1,000 ms (inclusive; 9 equal steps) and $S\in {\left\{s_{n}\right\}}_{n=0}^{40}=\left\{\;s_{0},\ldots ,s_{40}\right\}$ represents SSIs between 0 and 1 (inclusive; 40 equal steps). The histogram of the occurrence of all $\left(t_{m},\;s_{n}\right)$ pairs was normalized by the number of total trials (4,000) to form the joint probability of *T* and *S*, p(T, S) (Figure 4a). $p\left(T=t_{m},\;S=s_{n}\right)$ represents the probability of each $\left(t_{m},\;s_{n}\right)$ pair, that is, the probability of $S=s_{n}$ and $T=t_{m}$. Note that $p\left(T=t_{m},\;S=s_{n}\right)=p\left(S=s_{n},\;T=t_{m}\right)$ (Ross, 2014).

The probability of $S=s_{n}$ under the condition $T=t_{m}$, or the probability of an SSI, $s_{n}$, conditioned on a temporal offset, $t_{m}$, is defined by (Ross, 2014):

(1) $$p\left(S=s_{n}|T=t_{m}\right)=\frac{p\left(S=s_{n},\;T=t_{m}\right)}{p\left(T=t_{m}\right)}$$

In the above conditional probability, SSI is variable while the temporal offset is fixed at $T=t_{m}$. That is, to find the probability of SSIs assessed in the trials with an offset of $t_{m}$, we normalize the joint probability $p\left(S=s_{n},\;T=t_{m}\right)$ by $p\left(T=t_{m}\right)$. Since $p\left(T=t_{m}\right)=0.1$, m=1, …, 10, $p\left(S=s_{n}|T=t_{m}\right)=p\left(S=s_{n},\;T=t_{m}\right)/0.1$ (thin curves in Figure 4b).

The conditional cumulative distribution of $p\left(S|T=t_{m}\right)$, $F\left(S\leq s_{n}|T=t_{m}\right)$, is defined by (Ross, 2014):

(2) $$F\left(S\leq s_{n}|T=t_{m}\right)=\sum_{\forall s_{i}\leq s_{n}}p\left(S=s_{i}|T=t_{m}\right).$$

Note that if $s_{n}=1$, $F\left(S\leq s_{n}|T=t_{m}\right)=\sum_{\forall s_{i}\leq s_{n}}p\left(S=s_{i}|T=t_{m}\right)=1$.

The probability of SSIs assessed in all trials across subjects conditioned on a temporal offset, $t_{m}$, is $p\left(S|T=t_{m}\right)$. The *k*‐means algorithm was then used to cluster the $p\left(S|T=t_{m}\right)$ curves ( m=1, …, 10) into three groups (Lloyd, 1982; see Discussion). The squared Euclidean distance was used to compute the distance from a $p\left(S|T=t_{m}\right)$ curve to a cluster center. The *k*‐means algorithm was repeated for 1,000 times to ensure the stability of clustering results.

Assume $\tau =\left\{\;t_{1},\ldots ,t_{j}\right\}$ are clustered in the same group after *k*‐means clustering. The probability of the temporal offsets in this group is $p\left(T\in \tau \right)=\sum_{\forall t_{p}\in \tau }p\left(T=t_{p}\right)$, and the joint probability of all $\left(t_{p},\;s_{n}\right)$ pairs in this group is $p\left(S=s_{n},\;T\in \tau \right)=\sum_{\forall t_{p}\in \tau }p\left(S=s_{n},\;T=t_{p}\right)$. Hence, the within‐group conditional probability $p\left(S=s_{n}|T\in \tau \right)$ and cumulative distribution $F\left(S\leq s_{n}|T\in \tau \right)$ can be respectively defined by (thick curves in Figure 4b and c):

(3) $$p\left(S=s_{n}|T\in \tau \right)=\frac{p\left(S=s_{n},\;T\in \tau \right)}{p\left(T\in \tau \right)}=\frac{\sum_{\forall t_{p}\in \tau }p\left(S=s_{n},\;T=t_{p}\right)}{\sum_{\forall t_{p}\in \tau }p\left(T=t_{p}\right)},$$

and

(4) $$F\left(S\leq s_{n}|T\in \tau \right)=\sum_{\forall s_{i}\leq s_{n}}p\left(S=s_{i}|T\in \tau \right).$$

Note that if $s_{n}=1$, $F\left(S\leq s_{n}|T\in \tau \right)=1$.

### 2.4 fMRI experiment

#### 2.4.1 Experimental design and stimuli

Eleven subjects participated in an fMRI experiment consisting of four 480‐s functional scans and three additional scans (see Section 2.4.2). The same visual stimuli used in the psychophysical experiment were projected from an LCD projector (Dell 3300MP) onto a 39‐cm wide region on a direct‐view screen mounted 15 cm in front of the subject's face, yielding the same horizontal field of view as that in the psychophysical experiment (Figure 1a, right). The subject lay supine with his/her head tilted forward, which was firmly supported and constrained by foam padding in the head coil. The simulated looming balls on the direct‐view wide‐field screen were experienced as if they were real objects passing by the subject's face. Tactile stimuli with the same intensity and duration as those in the psychophysical experiment were delivered to either side of the face via a flexible hose mounted to the base of the MR‐compatible apparatus (Figure 1a, right). The subject manually adjusted each nozzle to precisely align the air puff with the looming ball in repeated calibration trials before the scanning.

Each functional scan contained 40 randomized trials (4 event types × 2 sides × 5 occurrences) with tactile‐only (T), visual‐only (V), tactile‐visual out‐of‐sync (TVoS), or tactile‐visual in‐sync (TViS) stimuli delivered to either side of the face (Figures 1b and 2b). The inter‐trial interval was randomized between 10 and 14 s (average = 12 s). The subject maintained central fixation during the entire scan, and made no response to each unisensory (T or V) event. In each TVoS event, an air puff was delivered at 100 ms following the onset (0 ms) of a looming ball on the same side, which was the direct superposition (spatial summation) of unisensory events in two different modalities (T and V) (Figure 2b). Following the onset of a looming ball in each TViS event, an air puff was delivered to the same side with an optimal temporal offset individually estimated for each subject from the psychophysical experiment (see Supporting Information Table S1). In each multisensory event, the subject maintained central fixation and reported the perceived event type by pressing a button (left: TVoS; right: TViS) on an MR‐compatible response pad (Current Designs Inc.) under the right hand. The subject's accuracy of response was recorded on the same stimulus computer.

#### 2.4.2 Image acquisition

Subjects were scanned with an 8‐channel head coil in a General Electric MR750 3‐T MRI scanner at the Center for Functional MRI at UCSD. In each fMRI session, four functional scans were acquired by an echo‐planar imaging sequence (single‐shot EPI; bandwidth = 62.5 kHz; flip angle = 60°; TE = 30.1 ms; TR = 1,000 ms; field of view = 224 × 224 mm; matrix = 64 × 64; voxel size = 3.5 × 3.5 × 3.5 mm; 19 axial slices; 480 TR per volume after discarding 8 dummy TRs). Two field map scans were acquired with the same orientation and dimensions as the functional scans. Lastly, an alignment scan was acquired by a fast spoiled gradient‐echo sequence (FSPGR; field of view = 256 × 256 mm; matrix = 256 × 256; voxel size = 1 × 1 × 1.3 mm; 106 axial slices) at the same volume center and orientation as the functional images. On a different day, two sets of high‐resolution structural images (FSPGR; field of view = 256 × 256 mm; matrix = 256 × 256; voxel size = 1 × 1 × 1 mm; 160–170 axial slices) were acquired for each subject.

#### 2.4.3 fMRI data analysis

Geometric distortions in functional images were corrected using two field map scans and post‐processing files from http://fmri.ucsd.edu/Howto/3T/fieldmap.html. For each subject, four field‐map corrected functional scans (480 time points per scan) were concatenated and then motion‐corrected using *3dvolreg* in the Analysis of Functional NeuroImages (AFNI) software package (Cox, 1996). For each subject, cortical surfaces were reconstructed from the average of two sets of high‐resolution structural images using the FreeSurfer software package (Dale, Fischl, & Sereno, 1999; Fischl, Sereno, & Dale, 1999a). Functional images were registered with cortical surfaces by manual blink comparison. An initial transformation matrix was obtained by registering the T1‐weighted alignment scan to the structural images used to make the surface. This was then refined by direct registration of the functional to structural images. The concatenated time series of fMRI signal in each voxel (1,920 time points) was analyzed using AFNI *3dDeconvolve* tool (Ward, 2002) with the following regressors in the general linear model (GLM): (1) baseline trends consisting of constant, linear, and quadratic drifts; (2) motion parameters (six degrees of freedom) obtained from the output of AFNI *3dvolreg*; and (3) a stimulus time series (event onset = 1; non‐event period = 0) for each of eight distinct event types (Left‐face stimuli: T_*L*_, V_*L*_, TVoS_*L*_, and TViS_*L*_; Right‐face stimuli: T_*R*_, V_*R*_, TVoS_*R*_, and TViS_*R*_; each occurred 20 times in 1,920 s). The maximum time lag for estimating the hemodynamic response function was set to 8 TR (8 s). The GLM analysis yields a partial *F*‐statistic *F*_(1,1798)_ and a hemodynamic response curve for each event type. Each *F*‐statistic value of a voxel was then multiplied by a sign indicating the direction of signal change (positive or negative BOLD signals), which was determined by summing the area under the estimated hemodynamic response curve (Chevrier, Noseworthy, & Schachar, 2007). The resulting signed *F*‐statistics were rendered on inflated cortical surfaces of each individual subject using FreeSurfer.

#### 2.4.4 Surface‐based group average

**Figure 5:**
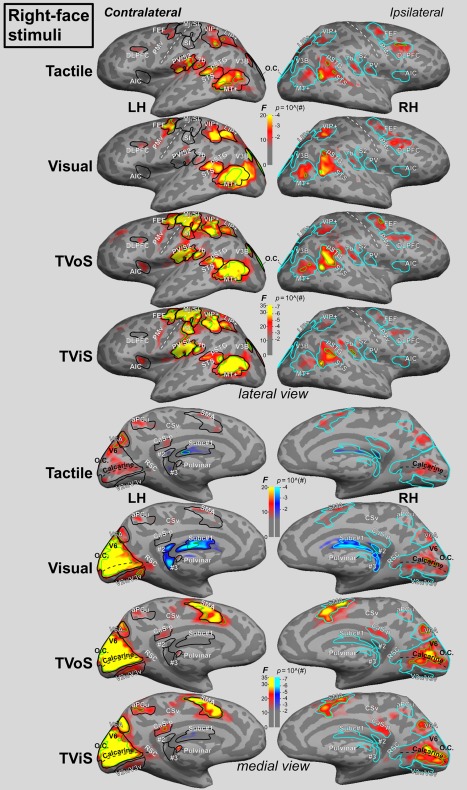
Group‐average statistical maps in response to right‐face stimuli in the fMRI experiment. Black contours in the left hemisphere (LH): sROIs outlined in response to right‐face stimuli (Figure 5). Cyan contours in the right hemisphere (RH): sROIs outlined in response to left‐face stimuli (Figure 6). Green contours in both hemispheres: brain regions with significant activations across 11 subjects (*t*_(10)_ > 2.76, one‐tailed; *p* < .01, uncorrected). Tactile and visual maps use one color scale, and TVoS and TViS maps use another color scale. Yellow‐red color bar: activation. Cyan‐blue color bar: deactivation. CSv: cingulate sulcus visual area (Smith et al., 2012). RSC: retrosplenial cortex (Huang & Sereno, 2013). Other abbreviations as in text [Color figure can be viewed at http://wileyonlinelibrary.com]

**Figure 6:**
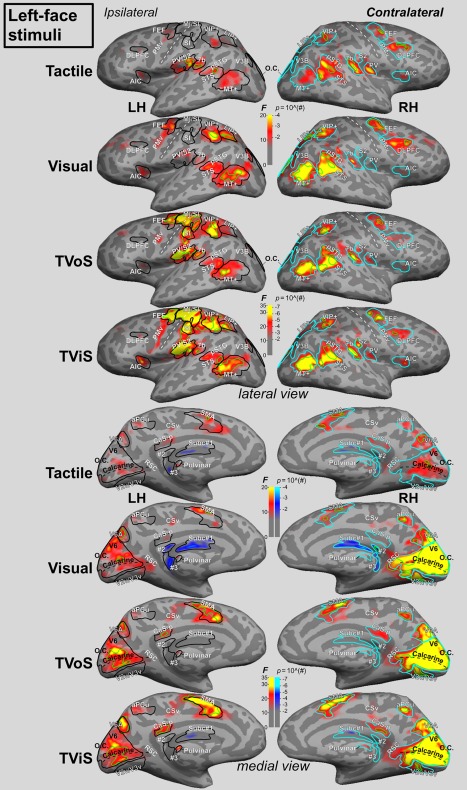
Group‐average statistical maps in response to left‐face stimuli in the fMRI experiment. All conventions follow Figure 5 [Color figure can be viewed at http://wileyonlinelibrary.com]

Spherical‐averaging methods were used to obtain surface‐based group‐average maps of signed *F*‐statistics for each event type (Fischl, Sereno, Tootell, & Dale, 1999b; Hagler, Riecke, & Sereno, 2007). For each subject, the cortical surface of each hemisphere was inflated into a sphere and registered with an average sphere using a sulcus‐based criterion (FreeSurfer *mris_register*). The *F*‐statistic map on each individual cortical surface was then interpolated to the average sphere using FreeSurfer *mri_surf2surf*. The resulting *F*‐statistic maps of the same event type were averaged across subjects (*n* = 11) in the common spherical coordinate system, and subsequently back‐sampled onto the cortical surfaces of a representative subject (Figures 5 and 6). Note that by using a thoroughgoing surface‐based pipeline, where 3D fMRI data is sampled to individual subject surfaces as a first step, we avoid the need for large 3D smoothing (blurring) kernels in the cross‐subject averaging step, yet produce better cross‐subject alignment than is possible using standard 3D volume‐based averaging methods. The result is that highly significant peak responses are better preserved and better aligned. In each of the group‐average maps, areas showing high average *F*‐statistic values suggest that they are highly significant across subjects and have a high degree of sulcal alignment across individual cortical surfaces, which were then validated using a *t*‐test (one‐tailed) at each vertex on the average spherical surface (see green contours in Figures 5 and 6).

**Figure 7:**
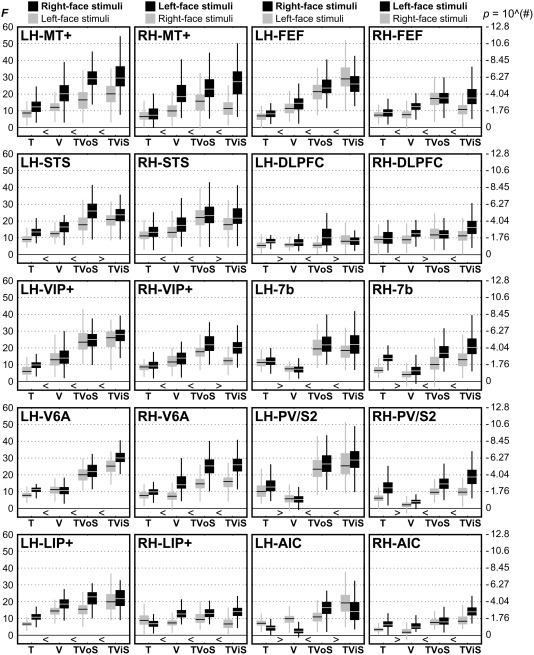
Box plots of group‐average *F*‐statistic distributions in response to unisensory and multisensory event types in ten bilateral sROIs (see Figures 5 and 6). For each sROI, black and gray box plots respectively indicate the responses to contralateral and ipsilateral stimuli. The top and bottom of each box respectively represent *F*_Q3_ and *F*_Q1_. The mark within each box indicates median. The height of each box indicates IQR (*F*_Q3_ − *F*_Q1_). Each whisker indicates 1.5 IQR above or below the box. Each inequality symbol compares the *F*_Q3_ values between two adjacent event types (contralateral stimuli only; see Supporting Information Tables S3 and S4). Each *F*‐statistic value on the left y‐axis corresponds to a *p*‐value (Bonferroni‐corrected, n = 11) estimated using a corresponding value (#) on the right y‐axis, for example, *p* = 10^(−1.76)^ = 0.0174

**Figure 8:**
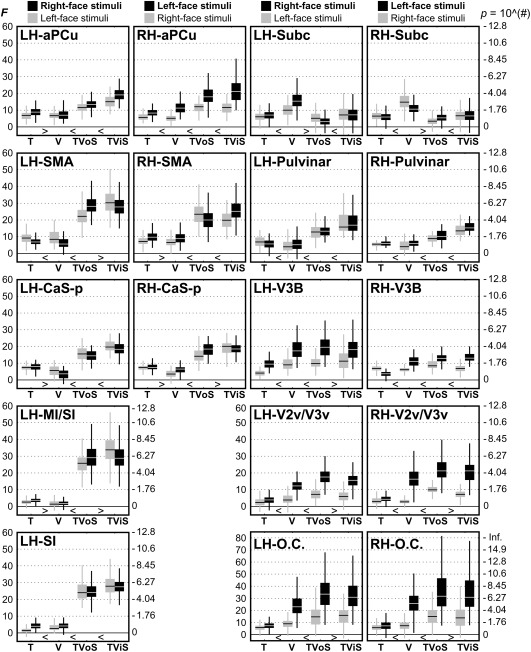
Box plots of group‐average *F*‐statistic distributions in response to unisensory and multisensory event types in eight bilateral and two unilateral sROIs (see Figures 5 and 6). Note that a different scale is used for the occipital cluster (O.C.). Other conventions follow Figure 7

To compare the group‐average statistical responses to different event types, we defined a set of surface‐based regions of interest (sROIs) with fixed contours on the same cortical surfaces (Figures 5 and 6). In the present study, we assume that most areas have a preference for contralateral stimuli. Therefore, the contours of sROIs on the left hemisphere (black contours in Figure 5) were outlined based on activations driven by right‐face stimuli, and vice versa. By gradually increasing the statistical threshold in the map of each event type, an interim sROI was identified as a region detached from its neighboring activations, and then outlined automatically using a surface‐based flood‐fill algorithm (csurf *tksurfer*). The final sROI contour was obtained by merging the contours of all interim sROIs across event types. Therefore, some of the final sROI contours do not match the exact extent of activations at a fixed statistical threshold in maps of different event types (Figures 5 and 6). Finally, the vertices enclosed in each final sROI were extracted, and the distribution of *F*‐statistic values associated with these vertices was shown in a box plot for each of eight event types (Figures 7 and 8).

## 3 RESULTS

### 3.1 Psychophysical experiment

Results of the psychophysical experiment are first illustrated in detail for a representative subject (Figure 3a). Each box plot (Figure 3a; left column) shows the median and IQR (the difference between the upper and lower quartiles; IQR = Q3 − Q1) of SSIs in 20 trials at each temporal offset (10 left‐face and 10 right‐face trials combined). Within each subject, no significant difference is found between the average SSI of the left‐face trials and that of the right‐face trials at each temporal offset (two‐tailed *t*‐test, *p* > .05, Bonferroni‐corrected; data not shown). As the temporal offset between the visual and tactile stimuli increases, the SSI‐median and SSI‐mean curves both rise steadily, peak at 900 ms, and then decrease at 1,000 ms. The SSI‐IQR and SSI‐s.d. curves both rise with the temporal offset, peak at 700 ms, and then decrease to reach a local minimum at 900 ms (as indicated by arrows in Figure 3a).

The ascending trends in the SSI‐median and SSI‐mean curves are consistent across 20 subjects, but they vary in the temporal offset where the peak occurs (Figure 3; Supporting Information Figures S2 and S3). In the single‐subject SSI‐median curves, the first peak occurs at 800, 900, and 1,000 ms in 2, 8, and 10 subjects, respectively (Figure 3; Supporting Information Figure S2). In the single‐subject SSI‐mean curves, the first peak occurs at 800, 900, and 1,000 ms in 2, 8, and 10 subjects, respectively (Figure 3; Supporting Information Figure S3; note that these subjects are slightly different from those in the SSI‐median curves). Among the curves peaking at 800 or 900 ms, only Subjects 1, 12, 19 show significant decreases (two‐tailed *t*‐test, *p* < .01, Bonferroni‐corrected) passing the peak in both the SSI‐median and SSI‐mean curves (Figure 3; Supporting Information Figures S2 and S3). Group‐average curves (*n* = 20) of single‐subject SSI‐median and SSI‐mean curves show insignificant decreases passing the peak at 900 ms (Figure 3b, top row). In sum, the single‐subject and group‐average curves show that SSI increases with the temporal offset and peaks between 800 and 1,000 ms. In about half of the subjects, SSI decreases passing the peak at 800 or 900 ms, suggesting that an air puff delivered shortly after the looming ball disappeared (Figure 2a, bottom) was perceived as slightly out of sync with the ball (see Discussion).

Group‐average curves (*n* = 20) of single‐subject SSI‐IQR and SSI‐s.d. curves show that they both increase with the temporal offset, peak at 600 ms, decrease to reach a local minimum at 900 ms, and then slightly rebound at 1,000 ms (Figure 3b, bottom row). While the profiles of individual SSI‐IQR and SSI‐s.d. curves are variable across subjects (Supporting Information Figures S2 and S3), most of them show a local maximum between 500 and 700 ms and a local minimum between 800 and 900 ms. The local maxima in SSI‐IQR and SSI‐s.d. curves suggest that subjects exhibited elevated uncertainty in assessing the synchrony of multisensory stimuli at these offsets. The offsets of the local minima in SSI‐IQR and SSI‐s.d. curves coincide with those of the peaks in SSI‐median and SSI‐mean curves, suggesting that optimally in‐sync multisensory stimuli were perceived and assessed with a low level of uncertainty.

To determine whether the subject's variability (uncertainty) in assessing the synchrony of multisensory stimuli is correlated with SSI, we further computed the correlation coefficients (*r*) between 20 pairs of SSI‐median and SSI‐IQR curves and between 20 pairs of SSI‐mean and SSI‐s.d. curves at each temporal offset (Figure 3b). The resulting *r* curves (Figure 3c) can be approximately divided into four phases: (1) at offsets between 100 and 200 ms (out‐of‐sync stimuli), subjects who assessed the stimuli with lower SSIs also exhibited lower within‐subject variability (lower IQR and s.d.), yielding high positive correlation coefficients; (2) as the offset continues to increase, correlation coefficients decrease to reach zero between 600 and 700 ms (neither in‐sync nor out‐of‐sync stimuli), where the assessment of SSI is uncorrelated with the high within‐subject variability (high IQR and s.d.); (3) correlation coefficients then increase negatively with the increase of the offset before reaching the next phase; and (4) at offsets between 800 and 1000 ms (in‐sync stimuli), subjects who assessed the stimuli with higher SSIs also exhibited lower within‐subject variability (lower IQR and s.d.), yielding high negative correlation coefficients.

The results of behavioral data modeling shows the joint probability of *T* and *S*, p(T, S), which is the 2D histogram of the occurrence of each $\left(t_{m},\;s_{n}\right)$ pair normalized by the total number of trials (Figure 4a). Each row of the 2D histogram shows the trend of SSIs assessed at each offset, where *T* is fixed at $T=t_{m}$ while *S* varies; as expressed by $p\left(S|T=t_{m}\right)$. The rows with an offset less than 400 ms reach the peak at SSI = 0, while the rows with an offset more than 700 ms reach the peak at SSI = 1. The rows with an offset between 400 and 700 ms fluctuate without a clear tendency.

The *k*‐means algorithm clustered $p\left(S|T=t_{m}\right)$ curves into three groups, with the following ranges of temporal offsets (Figure 4b): (1) $\tau_{1}\in \left\{\;100,\;200,\;\text{and}\;300\;\text{ms}\right\}$ (1,200 trials total; mean SSI = 0.129 ± 0.145); (2) $\tau_{2}\in \left\{\;400,\;500,\;600,\;\text{and}\;700\;\text{ms}\right\}$ (1,600 trials total; mean SSI = 0.433 ± 0.27); and (3) $\tau_{3}\in \left\{\;800,\;900,\;\text{and}\;1,000\;\text{ms}\right\}$ (1,200 trials total; mean SSI = 0.879 ± 0.15). In Figure 4b, each thin curve represents $p\left(S|T=t_{m}\right)$ at a temporal offset, $t_{m}$, while each thick curve represents $p\left(S=s_{n}|T\in \tau \right)$ of each group. Figure 4c shows the curve of conditional cumulative distribution, $F\left(S=s_{n}|T\in \tau \right)$, for each group. More than 81% of the trials in Group 1 were assessed with SSI < 0.25, and more than 82% of the trials in Group 3 were assessed with SSI > 0.75. The trials in Group 2 show an approximately linear trend rising with SSI, without a bias toward either end.

### 3.2 fMRI experiment

In each fMRI session, the overall accuracy of response to multisensory events (TVoS and TViS on both sides) was measured from a total of 80 trails in four scans. The average accuracy was 97 ± 3% across 11 subjects, suggesting that they were highly attentive during the experiment.

Results of the fMRI experiment are illustrated by surface‐based group‐average maps (*n* = 11) of signed *F*‐statistics for each of eight event types (four in Figure 5 and four in Figure 6). Eighteen pairs of matching bilateral sROIs were outlined on inflated cortical surfaces. Two unilateral sROIs were outlined in the left primary sensorimotor cortex (hand/arm representations in MI/SI; finger representations in SI). Activations in most of the selected sROIs were repeatable and statistically significant across 11 subjects (*p* < .01, cluster corrected; green contours in Figures 5 and 6), particularly in response to multisensory event types. For each sROI in each hemisphere, we compare across eight event types the distributions of average *F*‐statistics associated with the vertices enclosed within the same contour (box plots in Figures 7 and 8). The area and number of vertices enclosed in each sROI are summarized in Supporting Information Table S2. The average *F*‐statistic value at the upper quartile (*F*_Q3_) of a box plot is selected to facilitate the comparison of statistical significance across event types (Supporting Information Table S3). For each sROI, we mainly compare *F*_Q3_ values in response to contralateral stimuli, as indicated by subscripts (L or R) in the following paragraphs. A corresponding *p*‐value is estimated from each *F*_Q3(1, 1,798)_, and then subjected to Bonferroni correction (*n* = 11) (Supporting Information Table S4).

#### 3.2.1 MT+

The sROI of MT+ complex was outlined as a large region encompassing middle temporal area (MT), dorsal aspect of the medial superior temporal area (MSTd), and fundus of the superior temporal area (FST) (Kolster, Peeters, & Orban, 2010). Both LH‐MT+ and RH‐MT+ showed higher *F*_Q3_ in response to contralateral stimuli (black boxes) than ipsilateral stimuli (gray boxes) in all event types (Figure 7). A sequential increase in *F*_Q3_ in response to contralateral stimuli is expressed by T_*R*_ < V_*R*_ < TVoS_*R*_ < TViS_*R*_ for LH‐MT+ (Figures 5 and 7); T_*L*_ < V_*L*_ < TVoS_*L*_ < TViS_*L*_ for RH‐MT+ (Figures 6 and 7).

#### 3.2.2 STS

An sROI located at the superior temporal sulcus was outlined and labeled STS (Beauchamp et al., 2004a, 2004b, 2008), which extends into the posterior superior temporal gyrus (pSTG) and into the middle temporal gyrus. Both LH‐STS and RH‐STS showed higher *F*_Q3_ in response to contralateral stimuli than ipsilateral stimuli in all event types (Figure 7). A sequential increase in *F*_Q3_ in response to contralateral stimuli is expressed by: T_*R*_ < V_*R*_ < TViS_*R*_ < TVoS_*R*_ for LH‐STS (Figures 5 and 7); T_*L*_ < V_*L*_ < TViS_*L*_ < TVoS_*L*_ for RH‐STS (Figures 6 and 7).

#### 3.2.3 VIP+

An sROI located at the confluence of the postcentral sulcus and anterior intraparietal sulcus was outlined and labeled VIP+ (plus sign indicates a complex with multiple subdivisions; Huang et al., 2017; Sereno & Huang, 2006, 2014). Both LH‐VIP+ and RH‐VIP+ showed higher *F*_Q3_ in response to contralateral stimuli than ipsilateral stimuli in all event types except TVoS (LH) (Figure 7). LH‐VIP+ showed a sequential increase in *F*_Q3_ in response to contralateral stimuli, as expressed by T_*R*_ < V_*R*_ < TVoS_*R*_ < TViS_*R*_ (Figures 5 and 7). RH‐VIP+ showed a sequential increase in *F*_Q3_ in a slightly different order: T_*L*_ < V_*L*_ < TViS_*L*_ < TVoS_*L*_ (Figures 6 and 7).

#### 3.2.4 V6A

An sROI located at the anterior bank of the superior parieto‐occipital sulcus (POS) was outlined and labeled V6A (Pitzalis et al., 2013; Pitzalis, Fattori, & Galletti, 2015). Both LH‐V6A and RH‐V6A showed higher *F*_Q3_ in response to contralateral stimuli than ipsilateral stimuli in all event types except V (LH) (Figure 7). A sequential increase in *F*_Q3_ in response to contralateral stimuli is expressed by: T_*R*_ < V_*R*_ < TVoS_*R*_ < TViS_*R*_ for LH‐V6A (Figures 5 and 7); T_*L*_ < V_*L*_ < TVoS_*L*_ < TViS_*L*_ for RH‐V6A (Figures 6 and 7).

#### 3.2.5 LIP+

An sROI located in the intraparietal sulcus (IPS) was outlined and labeled LIP+ (plus sign indicates a complex map with multiple subdivisions; Hagler et al., 2007; Huang & Sereno, 2018; Sereno & Huang, 2006, 2014). The location of LIP+ is consistent with the anterior subdivisions of the IPS‐x strip (IPS‐3 to IPS‐5; Konen & Kastner, 2008). Both LH‐LIP+ and RH‐LIP+ showed higher *F*_Q3_ in response to contralateral stimuli than ipsilateral stimuli in all event types except T (RH‐LIP+) (Figure 7). A sequential increase in *F*_Q3_ in response to contralateral stimuli is expressed by: T_*R*_ < V_*R*_ < TVoS_*R*_ < TViS_*R*_ for LH‐LIP+ (Figures 5 and 7); T_*L*_ < V_*L*_ < TVoS_*L*_ < TViS_*L*_ for RH‐LIP+ (Figures 6 and 7).

#### 3.2.6 FEF

An sROI located at the intersection of the precentral sulcus and the superior frontal sulcus was outlined and labeled FEF (frontal eye fields; Hagler & Sereno, 2006; Hagler et al., 2007; Huang et al., 2015). Neither LH‐FEF nor RH‐FEF showed a consistent preference for contralateral stimuli across all event types (Figure 7). A sequential increase in *F*_Q3_ in response to contralateral stimuli is expressed by: T_*R*_ < V_*R*_ < TVoS_*R*_ < TViS_*R*_ for LH‐FEF (Figures 5 and 7); T_*L*_ < V_*L*_ < TVoS_*L*_ < TViS_*L*_ for RH‐FEF (Figures 6 and 7).

#### 3.2.7 DLPFC

An sROI located at the dorsolateral prefrontal cortex was outlined and tentatively labeled DLPFC (Hagler & Sereno, 2006). Neither LH‐DLPFC nor RH‐DLPFC showed a consistent preference for contralateral stimuli across all event types (Figure 7). LH‐DLPFC showed a sequential increase in *F*_Q3_ in response to contralateral stimuli, in the following order: V_*R*_ < T_*R*_ < TViS_*R*_ < TVoS_*R*_ (Figures 5 and 7). RH‐DLPFC showed a sequential increase in *F*_Q3_ in a completely different order: T_*L*_ < TVoS_*L*_ < V_*L*_ < TViS_*L*_ (Figures 6 and 7).

#### 3.2.8 7b

An sROI located at the posterior lateral sulcus was outlined and tentatively labeled 7b for contralateral and ipsilateral responses to tactile stimulation on the face (Dong et al., 1994; Huang & Sereno, 2007, 2018; Ishida et al., 2010; Robinson & Burton, 1980b, 1980c). Note that area 7b also showed a lower level of response to visual stimulation (see Discussion). Both LH‐7b and RH‐7b showed higher *F*_Q3_ in response to contralateral stimuli than ipsilateral stimuli in all event types except V (LH) (Figure 7). A sequential increase in *F*_Q3_ in response to contralateral stimuli is expressed by: V_*R*_ < T_*R*_ < TVoS_*R*_ < TViS_*R*_ for LH‐7b (Figures 5 and 7); V_*L*_ < T_*L*_ < TVoS_*L*_ < TViS_*L*_ for RH‐7b (Figures 6 and 7).

#### 3.2.9 PV/S2

An sROI located at the upper bank of the posterior lateral sulcus was outlined and labeled PV/S2 (PV: parietal ventral somatosensory area; S2: secondary somatosensory “area”) for contralateral and ipsilateral responses to tactile stimulation on the face (Disbrow, Roberts, & Krubitzer, 2000; Disbrow, Litinas, Recanzone, Padberg, & Krubitzer, 2003; Hihara, Taoka, Tanaka, & Iriki, 2015; Huang & Sereno, 2007, 2018; Robinson & Burton, 1980a, 1980b, 1980c; see Discussion). Both LH‐PV/S2 and RH‐PV/S2 showed higher *F*_Q3_ in response to contralateral stimuli than ipsilateral stimuli in all event types except V (LH) (Figure 7). A sequential increase in *F*_Q3_ in response to contralateral stimuli is expressed by: V_*R*_ < T_*R*_ < TVoS_*R*_ < TViS_*R*_ for LH‐PV/S2 (Figures 5 and 7); V_*L*_ < T_*L*_ < TVoS_*L*_ < TViS_*L*_ for RH‐PV/S2 (Figures 6 and 7).

#### 3.2.10 AIC

An sROI located at the anterior insular cortex was outlined and labeled AIC (Billington et al., 2011; Calvert et al., 2001). LH‐AIC showed a sequential increase in *F*_Q3_ in response to contralateral stimuli, in the following order: V_*R*_ < T_*R*_ < TViS_*R*_ < TVoS_*R*_ (Figures 5 and 7); RH‐AIC showed a sequential increase in *F*_Q3_ in a slightly different order: V_*L*_ < T_*L*_ < TVoS_*L*_ < TViS_*L*_ (Figures 6 and 7).

#### 3.2.11 aPCu

An sROI located at the anterior part of the precuneus was outlined and labeled aPCu (anterior precuneus; Filimon, Nelson, Huang, & Sereno, 2009; Huang et al., 2015; Huang & Sereno, 2018), which overlaps with the precuneus motion area (PcM) activated by optic‐flow stimuli (Uesaki & Ashida, 2015; Wada, Sakano, & Ando, 2016). Note that the LH‐aPCu extends anteriorly into the ascending ramus of the posterior cingulate sulcus, and it may contain more than one area. Both LH‐aPCu and RH‐aPCu showed higher *F*_Q3_ in response to contralateral stimuli than ipsilateral stimuli in all event types (Figure 8). LH‐aPCu showed a sequential increase in *F*_Q3_ in response to contralateral stimuli, in the following order: V_*R*_ < T_*R*_ < TVoS_*R*_ < TViS_*R*_ (Figures 5 and 8). RH‐aPCu showed a sequential increase in *F*_Q3_ in a slightly different order: T_*L*_ < V_*L*_ < TVoS_*L*_ < TViS_*L*_ (Figures 6 and 8).

#### 3.2.12 SMA

An sROI located at the medial superior frontal gyrus was outlined and tentatively labeled SMA (supplementary motor area), which extends into the middle part of the cingulate sulcus/gyrus and may overlap with pre‐SMA. LH‐SMA showed a sequential increase in *F*_Q3_ in response to contralateral stimuli, in the following order: T_*R*_ < V_*R*_ < TViS_*R*_ < TVoS_*R*_ (Figures 5 and 8). RH‐SMA showed a sequential increase in *F*_Q3_ in a completely different order: V_*L*_ < T_*L*_ < TVoS_*L*_ < TViS_*L*_ (Figures 6 and 8).

#### 3.2.13 CaS‐p

An sROI located at the posterior callosal sulcus was outlined and labeled CaS‐p (Rosen, Stern, Michalka, Devaney, & Somers, 2016). LH‐CaS‐p showed a sequential increase in *F*_Q3_ in response to contralateral stimuli, in the following order: V_*R*_ < T_*R*_ < TVoS_*R*_ < TViS_*R*_ (Figures 5 and 8). RH‐CaS‐p showed a sequential increase in *F*_Q3_ in a slightly different order: V_*L*_ < T_*L*_ < TViS_*L*_ < TVoS_*L*_ (Figures 6 and 8).

#### 3.2.14 MI/SI

An sROI was outlined and labeled LH‐MI/SI for a region of contralateral hand/arm sensorimotor representations extending between the superior precentral and postcentral gyri, which is only present in the left hemisphere because of right‐hand button presses (Figures 5 and 6). LH‐MI/SI showed insignificant response to all unisensory event types and significant response to all multisensory event types (Figures 5, 6, and 8).

#### 3.2.15 SI (fingers)

An sROI extending from the postcentral gyrus into the postcentral sulcus in the left hemisphere was outlined and tentatively labeled LH‐SI (Figure 5). This sROI contains somatosensory representations of fingers, which adjoin face representation at the inferior postcentral gyrus/sulcus (Huang & Sereno, 2007, 2018; see Discussion for the absence of response in SI face representation). LH‐SI showed insignificant response to all unisensory event types and significant response to all multisensory event types (Figures 5, 6, and 8).

#### 3.2.16 Subc

A cluster of three sROIs located underneath the corpus callosum were outlined and tentatively labeled “Subc” (subcortical areas #1, #2, and #3; see Discussion). Both LH‐Subc and RH‐Subc showed significant deactivation in response to contralateral and ipsilateral visual‐only stimuli (V_*L*_ and V_*R*_), and marginal or insignificant deactivation to other event types (Figures 5, 6, and 8).

#### 3.2.17 Pulvinar

An sROI located at the medial subcortical surface was outlined and labeled “Pulvinar” (as the pulvinar nucleus of the thalamus; Billington et al., 2011). Only RH‐Pulvinar showed a consistent preference for contralateral stimuli across event types (Figure 8). A sequential increase in *F*_Q3_ in response to contralateral stimuli is expressed by: T_*R*_ < V_*R*_ < TVoS_*R*_ < TViS_*R*_ for LH‐Pulvinar (Figures 5 and 8); T_*L*_ < V_*L*_ < TVoS_*L*_ < TViS_*L*_ for RH‐Pulvinar (Figures 6 and 8).

#### 3.2.18 V3B

An sROI located at the dorsal occipital lobe was outlined and labeled V3B (Smith, Greenlee, Singh, Kraemer, & Hennig, 1998), which is detached from the main occipital cluster as described below. Both LH‐V3B and RH‐V3B showed higher *F*_Q3_ in response to contralateral stimuli than ipsilateral stimuli in all event types except T (RH) (Figure 8). LH‐V3B showed a sequential increase in *F*_Q3_ in response to contralateral stimuli, in the following order: T_*R*_ < V_*R*_ < TViS_*R*_ < TVoS_*R*_ (Figures 5 and 8). RH‐V3B showed a sequential increase in *F*_Q3_ in a slightly different order: T_*L*_ < V_*L*_ < TVoS_*L*_ < TViS_*L*_ (Figures 6 and 8).

#### 3.2.19 V2v/V3v

An sROI was outlined and labeled V2v/V3v for ventral occipital areas separated from the main occipital cluster defined below. Both LH‐V2v/V3v and RH‐V2v/V3v showed higher *F*_Q3_ in response to contralateral stimuli than ipsilateral stimuli in all event types (Figure 8). A sequential increase in *F*_Q3_ in response to contralateral stimuli is expressed by: T_*R*_ < V_*R*_ < TViS_*R*_ < TVoS_*R*_ for LH‐V2v/V3v (Figures 5 and 8); T_*L*_ < V_*L*_ < TViS_*L*_ < TVoS_*L*_ for RH‐V2v/V3v (Figures 6 and 8).

#### 3.2.20 Occipital cluster (O.C.)

A large sROI was outlined and labeled O.C. for a continuous occipital cluster including areas V1v, V1d, V2d, V3d, V3A, and V6 in occipital lobe (Pitzalis et al., 2015, 2006, 2013; Tootell et al., 1997). Both LH‐O.C. and RH‐O.C. showed higher *F*_Q3_ in response to contralateral stimuli than ipsilateral stimuli in all event types (Figure 8). A sequential increase in *F*_Q3_ in response to contralateral stimuli is expressed by: T_*R*_ < V_*R*_ < TViS_*R*_ < TVoS_*R*_ for LH‐O.C. (Figures 5 and 8); T_*L*_ < V_*L*_ < TViS_*L*_ < TVoS_*L*_ for RH‐O.C. (Figures 6 and 8).

## 4 DISCUSSION

### 4.1 Design of multisensory looming stimuli

Previous neurophysiological and neuroimaging studies on multisensory integration have considered the following factors in designing their experiments and stimuli: (1) stimulus modalities (visual, auditory, tactile, and so on); (2) semantic or content congruency across modalities (Beauchamp, 2005b; Beauchamp et al., 2004a, 2004b; Calvert, 2001); (3) spatial congruency (Macaluso & Driver, 2001, 2005) or precise alignment of receptive fields across modalities (Avillac et al., 2005, 2007; Dong et al., 1994; Duhamel et al., 1998; Graziano et al., 1994, 1997; Huang et al., 2017; Ishida et al., 2010; McCollum et al., 2012; Sereno & Huang, 2006); (4) directional congruency across modalities (e.g., looming or receding; Maier, Neuhoff, Logothetis, & Ghazanfar, 2004; Maier et al., 2008; Tyll et al., 2013); and (5) temporal synchrony across modalities (Avillac et al., 2007; Bushara, Grafman, & Hallett, 2001; Bushara et al., 2003; Calvert et al., 2001; Marchant et al., 2012). In the present study, spatially aligned and directionally congruent looming visual and tactile stimuli were delivered to either side of the face with a varying temporal offset in psychophysical and fMRI experiments. The visual stimuli (looming balls) were simulated in 3D virtual reality and projected onto a 2D screen (CRT or LCD projector), which was located right in front of the subject's face. Although not being presented stereoscopically, the “virtual” looming ball was experienced as if it was actually approaching and flying past the face. The sense of presence was further enhanced by an air puff delivered tangentially, not perpendicularly, to the cheek with an optimal temporal offset. It is important to note that the peripheral looming stimuli in the present study were not designed to induce a sense of imminent (head‐on) collision, which is induced by presenting a looming object centrally (e.g., Billington et al., 2011; Cappe et al., 2009; Maier et al., 2008; Tyll et al., 2013). Furthermore, an air blast to the whole face may be synchronized with a centrally located looming object to enhance multisensory sensation of a head‐on impact in future studies.

### 4.2 Psychophysical experiment

Temporal synchrony is one of the fundamental principles of multisensory integration (Burr et al., 2009; Bushara et al., 2001; Calvert, 2001; Calvert & Thesen, 2004; Vroomen & Keetels, 2010). In typical temporal‐integration experiments, paired multisensory stimuli (e.g., a flash and a beep) are presented for the same duration in each modality, with various stimulus onset asynchronies (SOAs) in different pairs. The subject determines whether the multisensory stimuli are simultaneous or not in a “simultaneity judgment” (SJ) task, or determines which unisensory stimulus comes first in a “temporal order judgment” (TOJ) task. While the neural processing time may be slightly different in each modality, multisensory stimuli are usually perceived as being maximally simultaneous when SOA is small but not necessarily zero (see Burr et al., 2009; Harrar & Harris, 2005, 2008; Vroomen & Keetels, 2010).

In the present study, multisensory stimuli delivered with similar onset times (offset = 100 ms) were subjectively perceived and interpreted as completely out of sync (Figure 3). This is because the paradigms, stimuli, tasks, and measures in the present study are fundamentally different from those in the conventional psychophysical studies on multisensory integration, as discussed below. First, most of the paired multisensory stimuli in typical psychophysical studies are brief impulses with the same duration (e.g., 50 ms), and they do not overlap with each other when the stimulus duration is shorter than the SOA (e.g., 100 ms). In the present study, however, the looming ball has a fixed duration of 933 ms, which always overlaps with the 100‐ms air puff delivered with a temporal offset between 100 and 900 ms (1,000 ms excluded; Figure 2a). The subject's task is to determine whether the wind (air puff) could have been caused by the looming ball (i.e., the same physical event), and thus they are subjectively perceived and interpreted as ‘in sync’ with each other. Second, looming objects are dynamic and have the potential of causing tactile impacts on an observer in the real world (Cléry et al., 2015; Neppi‐Modona et al., 2004; Poljac et al., 2006). Because it takes time for a looming object to travel from a distance, it is less likely for an observer to feel the tactile impact right at its onset or shortly after. In the present study, the onset (0 ms) of a looming ball prompts the subject to anticipate a tactile impact at a later time between 100 and 1,000 ms. Third, the subject moved a cursor on a scroll bar to assess the subjective synchrony of multisensory stimuli in the present study. The cursor location was converted into a subjective synchrony index (SSI) between 0 and 1, which is a continuous visual analog scale that differs from the two‐alternative forced choice (2AFC) in SJ and TOJ tasks. The use of a visual analog scale allows us to observe a gradual transition from out‐of‐sync to in‐sync multisensory stimuli as the temporal offset increases, and to measure the range of the subject's uncertainty (IQR/s.d.) in assessing SSI at different temporal offsets, which is not obtainable by 2AFC tasks.

Across 20 subjects, multisensory looming stimuli with a temporal offset of 100 ms were consistently assessed with the lowest SSI and with low IQR/s.d (uncertainty). This suggests that an air puff delivered in close temporal proximity to the onset of a looming ball was undoubtedly perceived as out of sync with it. On the contrary, an air puff delivered at the moment when the looming ball was passing by (900 ms) or just flying past (1,000 ms) the face was perceived as optimally in sync (peak SSI) and with low IQR/s.d (uncertainty). In about half of the subjects, an air puff delivered at 1,000 ms was perceived as slightly out of sync (lower than the peak SSI) and assessed with slightly elevated uncertainty (rebound from the local minimum in SSI‐IQR and SSI‐s.d. curves). Either timing is possible in the real world because an object moving through the air induces turbulence along its path. The exact time of aerodynamic impact on the observer's body surface depends on many factors, such as object size, shape, proximity, and speed. For example, it is possible for a pedestrian to feel the “wind” during or after the passing of a cyclist or a car. While these results contradict with the general notion that synchronous stimuli typically have temporally aligned (or close) onsets (King, 2005; Vroomen & Keetels, 2010), the perception of looming objects must also take into consideration the laws of physics in the real world. Therefore, a different set of more ecologically valid rules will need to be written for the spatiotemporal integration of multisensory looming signals in future studies.

The results of behavioral data modeling suggest that the conditional probability distribution of SSI (*S*) given a temporal offset $T=t_{m}$, $p\left(S|T=t_{m}\right)$, can be clustered into three groups (Figure 4b and c): (1) low SSIs and low IQR/s.d. between 100 and 300 ms, where the stimuli were perceived as out‐of‐sync and assessed with a low level of uncertainty; (2) moderate SSIs and high IQR/s.d. between 400 and 700 ms, where the stimuli were perceived as neither out‐of‐sync or in‐sync and assessed with a high level of uncertainty; and (3) high SSIs and low IQR/s.d. between 800 and 1, 000 ms, where the stimuli were perceived as in‐sync and assessed with a low level of uncertainty. Furthermore, the high correlation coefficients (between SSI‐median and SSI‐IQR curves or between SSI‐mean and SSI‐s.d. curves; Figure 3c) between 100 and 200 ms and between 800 and 1,000 ms also suggest that multisensory stimuli with these temporal offsets were assessed with a low level of uncertainty across subjects.

### 4.3 fMRI experiment

As an initial step of our analysis, we identified all possible brain regions (sROIs) involved in the processing of unisensory and/or multisensory looming stimuli in the fMRI experiment. The selected sROIs contain both statistically significant and spatially aligned activations in a moderate‐sized group of subjects (*n* = 11), as shown by spherical‐averaging methods and validated by a second‐level statistical analysis (*t*‐test). Each sROI was outlined with a fixed contour on the cortical surface, and within which we compared the distribution of group‐average *F*‐statistics across different event types (Figures 7 and 8). For example, the *F*_Q3_ values in MT+ increase in the following order: T < V < TVoS < TViS (Figure 7). While interpreting the results, however, it is important to keep in mind that the surface‐based group averaging method has some limitations. First, each sROI within a subject may contain more than one functional subdivision or multiple unisensory and multisensory patches (e.g., Beauchamp et al., 2004a; Jiang, Beauchamp, & Fine, 2015), which were all merged into a single sROI in single‐subject and group‐average maps in the present study. Second, each sROI outlined in the group‐average maps contains only the average statistics as well as the central tendency of sROI locations across subjects, but not the distributions of statistics and spatial extent of each single‐subject sROI. Third, the max‐criterion for comparing multisensory and unisensory responses within voxels (Beauchamp, 2005a; Calvert et al., 2001; Tyll et al., 2013) may be used to compare the group‐average *F*‐statistics within an sROI between event types, for example, TVoS > max(T, V) by *F*_Q3_ values. However, arithmetic operations of the super‐ or sub‐additivity and mean criteria cannot be directly applied to nonlinear *F*‐statistics. Together, multisensory integration at the levels of individual voxel, patch, and sROI within each subject will need to be analyzed in detail in future studies. Here, we discuss major findings based on the overall trends of group‐average statistics (*F*_Q3_) within and across sROIs as follows.

#### 4.3.1 Lateralized activations

Most unisensory and multisensory sROIs, including the occipital cluster (V1v, V1d, V2d, V3d, V3A, and V6), V2v/V3v, V3B, MT+, STS, VIP+, LIP+, V6A, and aPCu, consistently showed higher *F*_Q3_ in response to contralateral stimuli than ipsilateral stimuli across event types (with few exceptions, see Supporting Information Table S3). These results suggest a contralateral preference for processing lateralized looming stimuli, which has not been demonstrated in previous human fMRI studies using centrally located looming stimuli (e.g., Tyll et al., 2013). Among the sROIs with a contralateral preference, MT+, STS, VIP+, V6A, and occipital cluster, also showed slightly weaker but significant ipsilateral responses, particularly to multisensory stimuli (Figures 5, 6, 7, 8; Supporting Information Table S3). While area MST (part of the MT+ complex) has been shown to respond to ipsilateral stimuli (Dukelow et al., 2001; Huk, Dougherty, & Heeger, 2002; Smith, Wall, Williams, & Singh, 2006), the contralateral and ipsilateral selectivity in areas STS, VIP+, V6A, and aPCu is less clear and needs further investigation (Beauchamp et al., 2008; Huang et al., 2017; Pitzalis et al., 2015, 2013). From an ecological perspective, contralateral and ipsilateral responses may play complementary roles in helping an observer (as a whole entity) to detect and avoid an impending threat regardless of its potential impact on either side of the body.

#### 4.3.2 Sensorimotor activations

An air puff sweeping across one side of the cheek activated contralateral and ipsilateral face representations in somatosensory areas PV/S2 and 7b (Chen, Kreutz‐Delgado, Sereno, & Huang, 2017; Disbrow et al., 2000; Huang & Sereno, 2007, 2018), but not in primary somatosensory cortex (see discussion immediately following). Right‐hand button presses in response to multisensory stimuli activated a region of hand/arm representations in left primary sensorimotor cortex (sROI LH‐MI/SI), which was not activated in unisensory event types (Figures 5, 6, and 8). This region extended inferiorly along the postcentral gyrus into an sROI labeled LH‐SI, which contains finger representations adjoining face representation (not activated) at the inferior postcentral gyrus/sulcus. The absence of activation in bilateral SI face representation in the group‐average maps was likely because a single air puff (100 ms) does not result in sustained stimulation to primary somatosensory areas, which also contain small receptive fields (see similar results in Chen et al., 2017; Huang et al., 2012). In contrast, areas PV/S2 and 7b in somatosensory association cortex contain large receptive fields that integrate higher‐order information such as tactile and/or visual motion (Disbrow et al., 2003, 2000; Dong et al., 1994; Hihara et al., 2015; Planetta & Servos, 2012; Robinson & Burton, 1980a, 1980b, 1980c).

On the medial wall, an sROI tentatively labeled SMA (possibly overlapping pre‐SMA) showed stronger responses to multisensory stimuli than to unisensory stimuli, without a consistent contralateral preference across event types (Figures 5, 6, and 8). Bilateral activations in SMA in response to unisensory and multisensory stimuli suggest that SMA may be involved in more than motor responses, that is, the activation would have been predominantly limited to the left hemisphere (right‐hand button presses). Further studies are required to refine the subdivisions in the sROI SMA and investigate their functional roles in unisensory/multisensory processing.

#### 4.3.3 Inter‐sensory interaction

Tactile‐only stimuli weakly activated areas V1 and V6 of the occipital cluster, V3B, LIP+, and FEF (Figures 5, 6, 7, 8). While these areas have long been considered unisensory, recent studies have begun to demonstrate tactile or auditory activations in early visual cortex, particularly V1 (Chen et al., 2017; Ghazanfar & Schroeder, 2006; Martuzzi et al., 2007; Merabet et al., 2007; Murray et al., 2016; Romei, Murray, Cappe, & Thut, 2009). Real‐world events often generate signals in more than one sensory modality. For example, a car passing by an observer induces winds over the body surface, which is perceived as tactile motion. In the present study, it is possible that an air puff sweeping across the face resulted in attentional modulations in early visual areas in anticipating a looming ball (though it did not actually happen in the tactile‐only events). Similarly, tactile activations were observed in high‐level visual motion areas MT+, STS, VIP+, V6A, and aPCu (Figures 5, 6, 7, 8; Huang et al., 2015). While areas STS and VIP+ have been demonstrated to be multisensory, it remains controversial whether areas MT+, V6A, and aPCu can be considered multisensory based on their tactile responses. In particular, recent studies suggested that tactile activations in human MT+ complex (or more specifically, area MST) could result from visual imagery of tactile motion on a body part (Beauchamp et al., 2007; Chen et al., 2017; Huang & Sereno, 2007; Jiang et al., 2015).

Visual‐only stimuli weakly activated sROIs PV/S2 and 7b at the posterior lateral sulcus, with lower *F*_Q3_ values than those in response to tactile‐only stimuli (Figures 5, 6, 7). Single‐unit recording studies in monkeys have shown that these areas, generally included as part of higher level somatosensory cortex, respond to near‐body visual stimuli (Dong et al., 1994; Graziano, 2004; Hihara et al., 2015; Ishida et al., 2010; Robinson & Burton, 1980b, 1980c). Human neuroimaging studies have also shown somatosensory, visual, and/or vestibular activations at the posterior Sylvian fissure overlapping with the sROI 7b outlined in the present study (see PIC/PIVC in Billington & Smith, 2015; PIVC in Cardin & Smith, 2010; 7b in Chen et al., 2017; PIC in Frank, Baumann, Mattingley, & Greenlee, 2014; 7b in Hagen & Pardo, 2002; PIVC in Huang et al., 2015; 7b in Huang & Sereno, 2007; PIVC in Smith, Wall, & Thilo, 2012). A possible functional role of the converging multisensory representations in the posterior Sylvian region is to coordinate avoidance movements of the head and body in response to looming threats. Future high‐resolution fMRI studies using a combination of somatosensory, visual, auditory, and vestibular stimuli are required to clearly map the subdivisions (PV/S2, 7b, PIVC, PIC, and auditory cortex) and their functions in this region within subjects (Huang & Sereno, 2018).

#### 4.3.4 Spatial and temporal multisensory integration

The group‐average statistics in sROIs in response to different contralateral event types are compared in pairs (tactile vs. visual, unisensory vs. multisensory, and out‐of‐sync vs. in‐sync stimuli) and expressed by a series of inequalities as follows (see also Figures 7 and 8; Table 1 and Supporting Information Table S3). First, bilateral sROIs MT+, STS, VIP+, V6A, V3B, LIP+, FEF, occipital cluster, and V2v/V3v were more strongly activated by contralateral visual‐only (V) stimuli than by tactile‐only (T) stimuli, as expressed by T < V. Second, direct spatial superposition of visual‐only and tactile‐only stimuli on the same side (i.e., TVoS events that were spatially aligned but temporally out‐of‐sync; Figure 2b) further enhanced the responses in those sROIs, as expressed by V < TVoS. Third, the effect of spatial integration of multisensory stimuli, regardless of temporal synchrony, was assessed using a *max‐min‐criterion*: max(T, V) < min(TVoS, TVis), which is a variation of the *max‐criterion*: max(T, V) < TV (Beauchamp, 2005a; Calvert et al., 2001; Tyll et al., 2013). In the present study, responses to contralateral unisensory/multisensory stimuli in all cortical sROIs except RH‐DLPFC met the *max‐min‐criterion* (Table 1), which are consistent with previous findings of spatial multisensory integration in neurophysiological and neuroimaging studies (Avillac et al., 2007; Macaluso & Driver, 2001, 2005). Fourth, the responses in bilateral sROIs MT+, V6A, 7b, PV/S2, LIP+, FEF, and aPCu were further enhanced by temporally in‐sync stimuli rather than out‐of‐sync stimuli, as expressed by TVoS < TViS. This is consistent with previous studies showing that temporally aligned multisensory stimuli enhance neuronal activity or hemodynamic responses (Avillac et al., 2007; Calvert et al., 2001; Marchant et al., 2012). However, the responses in bilateral sROIs STS, occipital cluster, and V2v/V3v showed the opposite results, as expressed by TVoS > TViS. Additionally, sROIs VIP+, V3B, DLPFC, and SMA show asymmetric results between hemispheres. These variable results suggest that different areas may have different mechanisms underlying the temporal integration of multisensory stimuli. For example, an air puff delivered right after the appearance of a looming ball (in a TVoS event) may result in attentional modulation and enhance “visual” responses of the occipital cluster. On the contrary, the effect of multisensory enhancement on early visual areas may be less strong when an air puff was delivered near the end of a looming ball (in a TViS event). Nevertheless, it is important to note that the temporal resolution of fMRI, even with a TR of 1,000 ms in the present study, is insufficient to tell exactly when the multisensory enhancement takes effect with sub‐second precision. Taken together, bilateral sROIs MT+, V6A, LIP+, and FEF consistently showed a “staircase‐like” increase in their responses to contralateral tactile‐only, visual‐only, tactile‐visual out‐of‐sync, and tactile‐visual in‐sync stimuli, as expressed by T < V < TVoS < TViS (Figure 7; Table 1).

**Table 1:** Comparing *F*_Q3_ values in response to contralateral stimuli by inequalities for each sROI

sROI	LH/RH	T < V	V < TVoS	max(T, V) < min(TVoS, TViS)	TVoS < TViS	T < V < TVoS < TViS
MT+	LH	x	x	x	x	x
	RH	x	x	x	x	x
STS	LH	x	x	x		
	RH	x	x	x		
VIP+	LH	x	x	x	x	x
	RH	x	x	x		
V6A	LH	x	x	x	x	x
	RH	x	x	x	x	x
LIP+	LH	x	x	x	x	x
	RH	x	x	x	x	x
FEF	LH	x	x	x	x	x
	RH	x	x	x	x	x
DLPFC	LH		x	x		
	RH	x			x	
7b	LH		x	x	x	
	RH		x	x	x	
PV/S2	LH		x	x	x	
	RH		x	x	x	
AIC	LH		x	x		
	RH		x	x	x	
aPCu	LH		x	x	x	
	RH	x	x	x	x	x
SMA	LH	x	x	x		
	RH		x	x	x	
CaS‐p	LH		x	x	x	
	RH		x	x		
MI/SI	LH		x	x		
SI	LH	x	x	x	x	x
Subc	LH	x			x	
	RH	x			x	
Pulvinar	LH	x	x	x	x	x
	RH	x	x	x	x	x
V3B	LH	x	x	x		
	RH	x	x	x	x	x
V2v/V3v	LH	x	x	x		
	RH	x	x	x		
O.C.	LH	x	x	x		
	RH	x	x	x		

sROIs above and below the central divider: see Figures 7 and 8, respectively. T, tactile (air puff); V, visual (looming ball); TVoS, tactile‐visual out‐of‐sync; TViS, tactile‐visual in‐sync; x, an sROI meeting a criterion as defined by an inequality; O.C., occipital cluster; LH/RH, left/right hemisphere.

#### 4.3.5 Other cortical and subcortical sROIs

Other findings in the remaining cortical and subcortical sROIs are discussed as follows. First, bilateral sROIs AIC located in the anterior insula showed higher responses to multisensory than to unisensory stimuli (meeting the *max‐min‐criterion*). While the responses in AIC were not as strong as those in other unisensory and multisensory sROIs, it could be involved in making judgments about the timing (duration) of looming objects (approaching threats) and in detecting the temporal synchrony or asynchrony of multisensory stimuli (Billington et al., 2011; Bushara et al., 2001, 2003; Calvert et al., 2001; Mobbs et al., 2010; Schienle, Wabnegger, Leitner, & Leutgeb, 2017). Second, bilateral sROIs CaS‐p located at the posterior callosal sulcus showed higher responses to multisensory than to unisensory stimuli. Activations in this region may result from increased attention in multisensory events, as it was suggested that CaS‐p supports the interaction between memory retrieval and attention (Rosen et al., 2016). Third, bilateral “Subc” clusters located underneath the corpus callosum showed stronger deactivation in response to visual‐only stimuli than tactile‐only and multisensory stimuli. These results were unlikely due to motion artifacts because of no motor response involved and because they were spatially restricted and consistently observed across individual subjects. Deactivation near the corpus callosum in response to optokinetic stimulation has been demonstrated previously (Dieterich, Bense, Stephan, Yousry, & Brandt, 2003). Further studies are needed to determine why passive observation of looming objects induced deactivation in this region. Finally, bilateral sROIs Pulvinar showed a staircase‐like increase in their responses to contralateral unisensory and multisensory stimuli, as expressed by T < V < TVoS < TViS. Activations in the pulvinar nucleus of the thalamus are consistent with a previous study suggesting that it is involved in low‐level detection of looming (Billington et al., 2011).

### 4.4 Future directions

One of the major challenges to study multisensory integration is that there are numerous possible combinations of stimulus factors and conditions that are multiplied, not just summed, across sensory modalities. In this initial study, there were 20 conditions in the psychophysical experiment and eight event types in the fMRI experiment, which were then multiplied by the minimum number of repetitions (trials) needed to reach statistical significance. Because of time constraints (and cost of MRI hours), it is impractical to test all kinds of stimulus combinations with unlimited repetitions in a single study. The experimental designs and stimulus characteristics in the present study can be expanded in the following directions. First, in future psychophysical experiments, a step of 50 ms or less can be used to refine the search of the optimal temporal offset between 800 and 1,200 ms (including the period briefly after the ball disappears). Second, in future fMRI experiments, multisensory stimuli with a temporal offset between 500 and 700 ms (neither in‐sync nor out‐of‐sync stimuli) can be used to study the neural basis of elevated uncertainty in assessing the synchrony of multisensory stimuli. Third, a varying spatial offset can be introduced between temporally aligned looming visual and tactile stimuli in both psychophysical and fMRI experiments. Without changing the locations of stimuli in one modality, spatially incongruent multisensory stimuli can be generated by varying the looming ball's expected points of impact on the face (Cléry et al., 2015; Neppi‐Modona et al., 2004; Poljac et al., 2006), or by delivering air puffs to different locations on the face via a wearable grid (Chen et al., 2017; Huang et al., 2012). Fourth, the traveling directions (looming or receding) of stimuli can be varied in each sensory modality to study the effect of directional congruency (Maier et al., 2008, 2004; Tyll et al., 2013).

In monkey neurophysiological experiments, bimodal neurons were found to respond to aligned visual and tactile stimuli presented near/on the face or other body parts (Avillac et al., 2007, 2005; Duhamel et al., 1998; Fogassi et al., 1996; Graziano et al., 1997, 1994; Hihara et al., 2015; Ishida et al., 2010). To map multisensory areas in humans, however, it is very challenging to set up similar experiments in the MRI scanner. In typical multisensory fMRI experiments, the subject indirectly views visual stimuli on a back‐projection screen via a mirror, which are spatially congruent (i.e., on the same side of the body) but not directly aligned with the tactile stimuli delivered to a body part (e.g., Jiang et al., 2015). In the present study, the direct‐view screen and flexible hoses were specifically designed to deliver spatially aligned looming visual and tactile stimuli immediately near the face (Figure 1). To study brain regions that respond to objects (threats) approaching the hand or foot (e.g., De Haan et al., 2016; De Paepe et al., 2016; Mobbs et al., 2010), looming visual stimuli can be projected onto a direct‐view screen near a body part, which are integrated with tactile stimuli delivered via a body‐part module of the wearable stimulation technology (Chen et al., 2017; Huang et al., 2012).

In the present study, brain activations in response to different event types were measured by an overall *F*‐statistic value estimated from the entire time series of each voxel. It is not possible to distinguish the temporal dynamics of activations between TVoS and TViS events at sub‐second resolution because: (1) all stimuli were delivered within one second of event onset (Figure 1b); (2) the change in hemodynamic response happens a few seconds later; and (3) functional images were acquired at a low temporal resolution (TR = 1,000 ms). In future studies, other neuroimaging techniques with higher temporal resolutions (e.g., EEG or MEG) can be used to investigate the different mechanisms and timing of multisensory processing of looming stimuli in low‐ and high‐level areas (Cappe et al., 2012; Vagnoni, Lourenco, & Longo, 2015). For example, MT+ showed responses to multisensory events as expressed by TVoS < TViS, but the occipital cluster showed the opposite response trend. The former could result from multisensory enhancement, while the latter could be accounted by top‐down attentional modulation or orienting in early visual areas.

### 4.5 Applications

Dynamic scenes of looming objects synchronized with looming sounds are commonly used in film, television, video games, virtual reality, and other media of entertainment to enhance the viewer's sense of presence (Wilkie & Stockman, 2012). The perception of objects apparently moving in depth is enhanced with the use of stereoscopic (3D) and wide‐field displays. To further enhance the sense of presence, physical effects in other sensory modalities, such as winds, scents, and seat motion, have been added to 3D films in limited theaters in theme parks or museums. For example, the audiences are being literally “touched” by monsters that apparently jump out of the screen. These multisensory effects collectively add an “extra dimension” to the existing 3D film, often referred to as the “4D” film (https://en.wikipedia.org/wiki/4D_film; IJsselsteijn, 2003; Neuendorf & Lieberman, 2010; Oh, Lee, & Lee, 2011). Although 4D film already exists for decades, little research has been done to investigate the underlying perceptual and neural mechanisms of these multisensory effects. The present study demonstrates a systematic framework to study the spatial and temporal integration of looming visual and tactile stimuli, which is one of the most common effects delivered in 4D theaters. Results in psychophysical and fMRI experiments provided direct scientific evidence to support the assumption that the strongest 4D effect takes place when visual and tactile stimuli are both spatially aligned and temporally synchronized. In the near future, the prototype of the multisensory stimulation apparatus demonstrated here can be expanded for developing the next generation immersive entertainment systems and media. The first step is to refine wearable devices to allow visual, tactile, and other modalities of stimulation to be delivered near the face with high spatial and temporal precision (Chalmers, Howard, & Moir, 2009, Chen et al., 2017; Huang et al., 2012; Huang & Sereno, 2012; Karns, Dow, & Neville, 2012). To put it in perspective, it would take a “Neurocinematic” approach (Hasson et al., 2008) and close collaboration among media content producers, device engineers, special effect programmers, experimental psychologists, and cognitive neuroscientists to produce and present multimedia that are effectively synchronized with multisensory effects.

## 5 CONCLUSION

A multisensory apparatus integrating a direct‐view wide‐field screen with flexible air hoses was designed and used to deliver spatially aligned looming visual and tactile stimuli near the face with a varying temporal offset. In the psychophysical experiment, multisensory stimuli presented with similar onset times (offset = 100 ms) were subjectively perceived and interpreted as completely out of sync and assessed with the lowest SSI. As the temporal offset increased, SSI increased steadily and then peaked between 800 and 1,000 ms, where multisensory stimuli were perceived as optimally in sync and assessed with a high certainty. These results suggest that the optimal temporal integration of looming visual and tactile stimuli took place at the moment of expected impact (on the face) rather than at stimulus onsets. In the fMRI experiment, sROIs were outlined in surface‐based group‐average statistical maps and most of them showed a preference for contralateral unisensory and multisensory stimuli. Intersensory activations were found in areas that are generally considered unisensory; for example, tactile response in V1. Statistical responses to different types of contralateral stimuli were compared by *F*_Q3_ values in each sROI. The responses to unisensory stimuli (T or V) were enhanced by the spatial summation of both (TVoS or TViS; regardless of temporal synchrony), as expressed by max(T, V) < min(TVoS, TViS). Temporally in‐sync (TViS) stimuli further enhanced the responses in bilateral sROIs MT+, V6A, 7b, PV/S2, LIP+, FEF, and aPCu, as expressed by TVoS < TViS. While this is consistent with the general principles of temporal multisensory integration, sROIs STS, occipital cluster, and V2v/V3v showed the opposite results. Finally, bilateral sROIs MT+, V6A, LIP+, and FEF showed staircase‐like responses, ascending from unisensory to multisensory stimuli: T < V < TVoS < TViS. In sum, this initial study demonstrated novel apparatus and methods for studying the spatiotemporal integration of multisensory looming stimuli near the face. There are numerous possible combinations of stimulus factors and conditions across modalities that can be explored in future studies. These studies will not only help to further understand the perceptual and neural mechanisms of multisensory integration but also provide a solid scientific foundation for developing the next generation multisensory entertainment systems and media, such as 4D film.

## Supporting information

Additional Supporting Information may be found online in the supporting information tab for this article.

Supporting Information
