# Supplementary material for: Spatiotemporal integration of looming visual and tactile stimuli near the face
Source: Hum Brain Mapp. 2018 Feb 6;39(5):2156–76. doi: 10.1002/hbm.23995 (PMC5895522; doi:10.1002/hbm.23995)
Supplement: Supplementary file 1 — Supporting Information [file HBM-39-2156-s001.pdf]

Spatiotemporal integration of looming visual and tactile stimuli near the face

Ruey-Song Huang[1*], Ching-fu Chen[2], Martin I. Sereno[3]

[1]Institute for Neural Computation and [2]Department of Electrical and Computer Engineering, University of California, San Diego, La Jolla, CA 92093, USA

[3]Department of Psychology and Neuroimaging Center, San Diego State University, San Diego, CA 92182, USA

Supporting Information

Supplementary Figures S1-S3

Supplementary Tables S1-S4

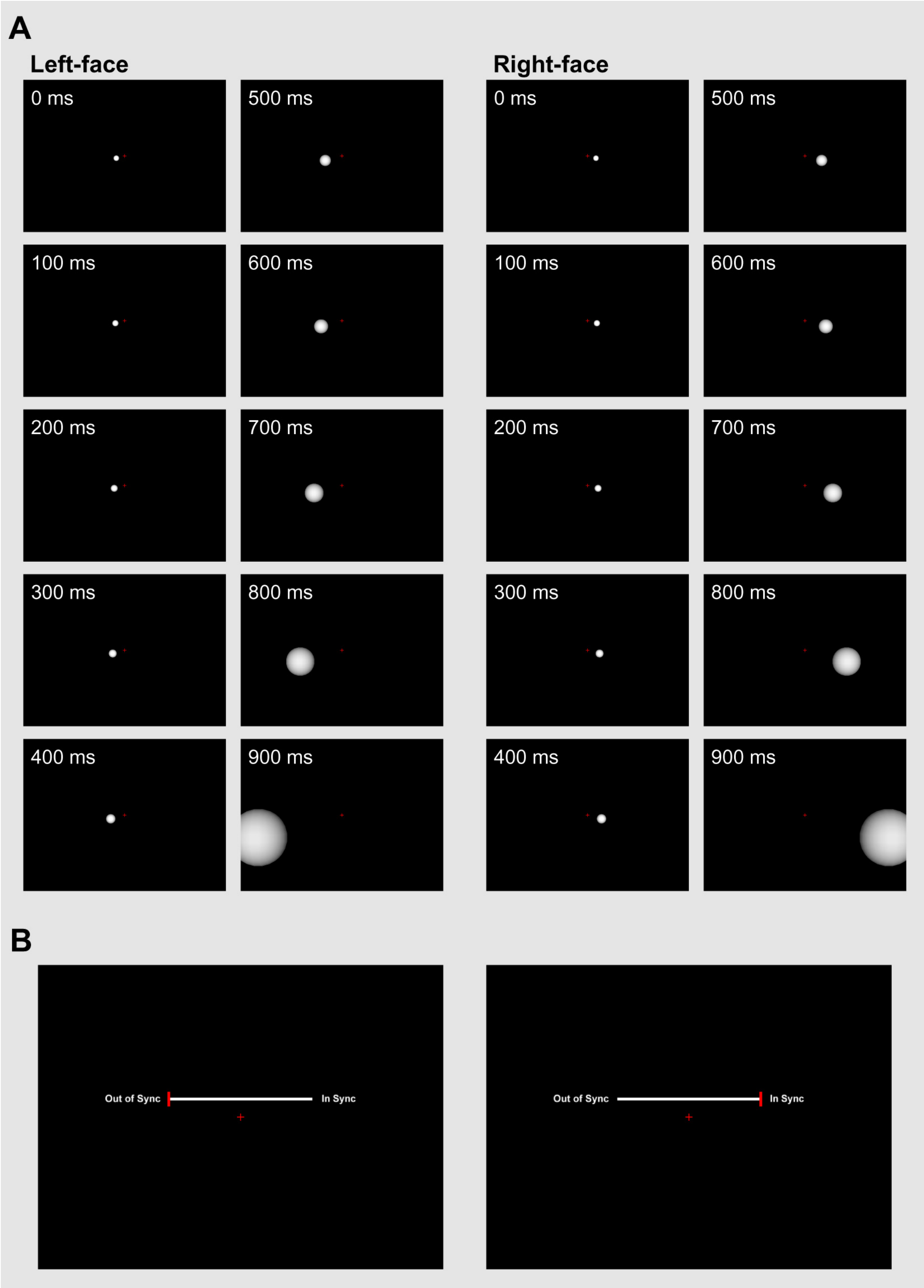

Figure S1. Screen shots of visual stimuli. (**A**) Key frames between 0-900 ms of a virtual looming ball near the left-face or right-face (in both fMRI and psychophysical experiments). (**B**) Examples of scroll bars shown in the psychophysical experiment.

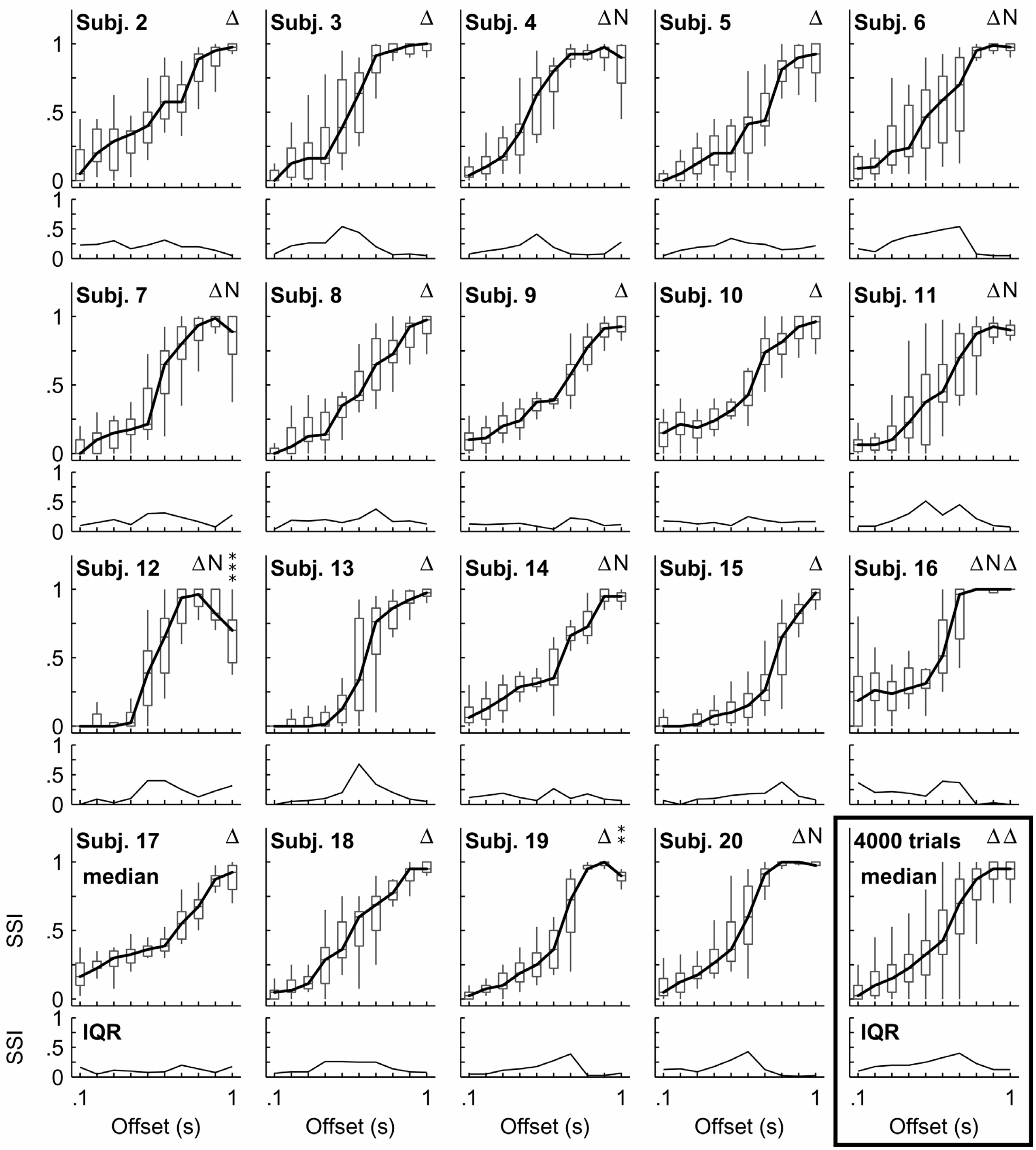

Figure S2. Single-subject SSI-median and SSI-IQR curves. Lower-right box: overall distributions of SSIs in 4000 trials across 20 subjects. Δ: peak. N: insignificant decrease from the peak. **: significant decrease from the peak, $p < 0.01$, corrected. ***: significant decrease from the peak, $p < 0.001$, corrected.

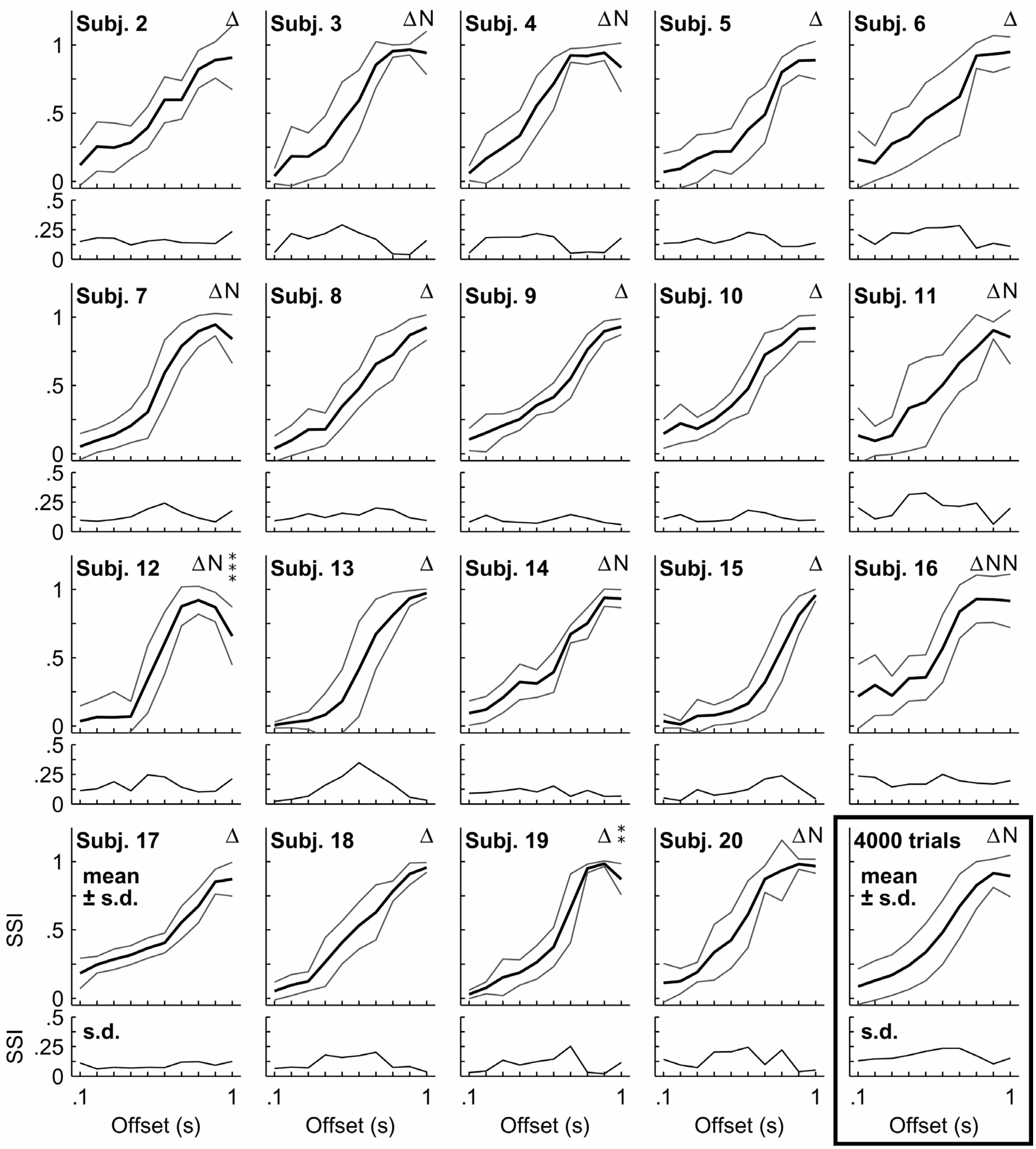

Figure S3. Single-subject SSI-mean and SSI-s.d. curves. All conventions follow Fig. S2.

Table S1. Estimated optimal temporal offset for each subject.

Subject	Average offset (ms) (left-face stimuli)	Average offset (ms) (right-face stimuli)	Estimated optimal temporal offset (ms)
1	880 ± 60	900 ± 44.7	890
2*	960 ± 49	970 ± 45.8	965
3*	900 ± 77.5	830 ± 119	865
4*	850 ± 92.2	900 ± 100	875
5*	960 ± 66.3	940 ± 66.3	950
6*	940 ± 66.3	890 ± 53.9	915
7*	850 ± 102	900 ± 63.2	875
8*	930 ± 64	950 ± 80.6	940
9*	960 ± 49	930 ± 64	945
10*	930 ± 64	950 ± 67.1	940
11*	850 ± 186	870 ± 90	860
12*	740 ± 66.3	860 ± 102	800
13	950 ± 67.1	930 ± 78.1	940
14	940 ± 49	940 ± 66.3	940
15	980 ± 40	960 ± 66.3	970
16	840 ± 102	890 ± 94.3	865
17	950 ± 67.1	950 ± 50	950
18	960 ± 49	950 ± 50	955
19	880 ± 40	870 ± 45.8	875
20	870 ± 78.1	890 ± 70	880

*: subjects also participated in the fMRI experiment.

Table S2. Surface area and vertices of each sROI.

	Left hemisphere (LH)		Right hemisphere (RH)	
sROI	Area (mm^2)	Vertices	Area (mm^2)	Vertices
MT+	1074.3	1795	823.0	1415
STS	405.6	577	935.5	1684
VIP+	735.1	1544	559.1	1074
V6A	271.2	550	575.3	1443
LIP+	367.2	604	568.7	933
FEF	526.6	948	647.6	1184
DLPFC	178.7	379	789.7	1783
7b	155.7	278	331.5	1005
PV/S2	628.1	1685	304.8	887
AIC	307.8	677	299.2	523
aPCu	359.6	577	249.6	440
SMA	656.1	959	708.9	1022
CaS-p	211.6	300	335.9	466
MI/SI	990.6	2010		
SI	268.1	533		
Subc	987.4	1530	615.8	1644
Pulvinar	52.7	310	90.6	171
V3B	198.2	390	232.6	413
V2v/V3v	888.1	1847	598.4	1448
O.C.	2762.5	6234	3243.2	6570

sROIs above/below the central divider: see Figs. 7/8. O.C.: occipital cluster.

Table S3. Upper quartile (F_{Q3}) of group-average F-statistic distributions in each sROI.

	Left hemisphere (LH)					Right hemisphere (RH)				
sROI	Stimuli	T	V	TVoS	TViS	Stimuli	T	V	TVoS	TViS
MT+	R-face	**15.5**	**25.3**	**33.5**	**36.6**	L-face	**11.4**	**25.4**	**28.8**	**33.5**
	L-face	10.5	14.5	21.2	24.9	R-face	9.29	13.3	19.7	15.1
STS	R-face	**15.3**	**19.3**	**30.4**	**27.1**	L-face	**16.5**	**22.1**	**28.5**	**27.6**
	L-face	10.9	14.1	22.1	23.4	R-face	13.7	16.5	26.4	21.8
VIP+	R-face	**11.4**	**18.4**	28.3	**30.8**	L-face	**11.7**	**17.2**	**27**	**23.5**
	L-face	8.79	16.9	**28.9**	29.8	R-face	10.2	15.3	20.2	14.4
V6A	R-face	**12.4**	12.8	**26**	**33.1**	L-face	**11.4**	**19.2**	**29.2**	**29.6**
	L-face	9.25	**13**	23.4	28.6	R-face	9.72	9.59	17.7	18.5
LIP+	R-face	**12.6**	**21.4**	**25.6**	**26.9**	L-face	8.49	**15**	**15.5**	**16.3**
	L-face	7.71	16.3	17.9	24.5	R-face	**11.8**	8.88	12.6	9.36
FEF	R-face	**10.3**	**17.4**	**28.6**	30.7	L-face	**10.8**	**14.5**	20.6	**23**
	L-face	8.46	14.2	25.5	**35.9**	R-face	9.15	9.71	**20.7**	13.4
DLPFC	R-face	**9.36**	**9.22**	**15.4**	10.3	L-face	**13.5**	**14.7**	14.6	**20.2**
	L-face	7.23	6.99	7.35	**11.6**	R-face	11.2	11.4	**15.5**	14.1
7b	R-face	**14.3**	8.85	**26.7**	**27.6**	L-face	**15.9**	**8.71**	**21.1**	**25.7**
	L-face	13.4	**9.13**	24.9	21.4	R-face	8.67	5.99	14.7	16.7
PV/S2	R-face	**16.6**	7.27	**31.7**	**34.1**	L-face	**15.5**	**5.1**	**17.8**	**23**
	L-face	13.7	**7.87**	28.8	33	R-face	7.65	3.46	11.9	12.2
AIC	R-face	5.78	4	**19.9**	19.8	L-face	**8.35**	**7.16**	**10.6**	**16.7**
	L-face	**8.74**	**11.6**	13.5	**24.1**	R-face	4.49	3.78	9.51	11.2
aPCu	R-face	**10.6**	**9.28**	**15.3**	**22**	L-face	**10**	**13.9**	**22.3**	**26.1**
	L-face	8.34	8.26	13.6	17.9	R-face	7.51	6.53	13.8	13.9
SMA	R-face	8.4	8.49	**32.4**	31.9	L-face	**11.9**	**11.5**	24	**29.8**
	L-face	**11.3**	**12.8**	26	**35.6**	R-face	9.03	8.63	**28**	23.7
CaS-p	R-face	**9.37**	5.63	17.1	21.4	L-face	**9.15**	**7.62**	**21.4**	20.7
	L-face	8.43	**7.99**	**18.9**	**22.9**	R-face	8.34	5.5	17.6	**21.9**
MI/SI	R-face	**4.58**	2.87	**34.4**	34.1					
	L-face	3.64	**3.03**	29.2	**39.5**					
SI	R-face	**5.56**	**5.61**	27.8	30.4					
	L-face	2.42	4.6	**28.9**	**32.2**					
Subc	R-face	**8.81**	**19.2**	5.06	10.3	L-face	7.61	13	**7.18**	**9.39**
	L-face	7.84	13	**7.88**	**10.8**	R-face	**8.3**	**18.5**	5	8.99
Pulvinar	R-face	7.71	**8.24**	15.5	22.7	L-face	**7.08**	**7.53**	**13.5**	**18.1**
	L-face	**9.62**	6.94	**15.8**	**23.1**	R-face	6.38	7.12	10.8	16.5
V3B	R-face	**11.6**	**22.6**	**24.1**	**22.7**	L-face	4.76	**13.5**	**14.8**	**15.5**
	L-face	5.44	12.3	12.5	15.9	R-face	**8.07**	7.12	11.2	8.36
V2v/V3v	R-face	**5.32**	**14.4**	**20.8**	**18.2**	L-face	**5.73**	**20.8**	**26.4**	**24.8**
	L-face	4.22	6.4	9.45	8.3	R-face	4.79	3.85	11.6	8.78
O.C.	R-face	**9.17**	**29.7**	**42.8**	**40.4**	L-face	**10**	**32.1**	**46.6**	**44.8**
	L-face	7.31	11.3	20.6	20.1	R-face	7.8	9.95	20.3	20.6

sROIs above/below the central divider: see Figs. 7/8. Shaded/unshaded: Contralateral/ipsilateral stimuli; O.C.: occipital cluster; Bolded number: the larger of the two F_{Q3} values in response to left-face and right-face stimuli under each event type.

Table S4. Estimated *p*-value for corresponding F_{Q3} in Table S3.

	Left hemisphere (LH)					Right hemisphere (RH)				
sROI	Stimuli	T	V	TVoS	TViS	Stimuli	T	V	TVoS	TViS
MT+	R-face	**9.49e-4**	**5.96e-6**	**9.04e-8**	**1.97e-8**	L-face	**8.10e-3**	**5.57e-6**	**9.80e-7**	**9.30e-8**
	L-face	1.32e-2	1.56e-3	4.99e-5	7.30e-6	R-face	2.57e-2	3.04e-3	1.08e-4	1.15e-3
STS	R-face	**1.04e-3**	**1.29e-4**	**4.53e-7**	**2.34e-6**	L-face	**5.55e-4**	**3.03e-5**	**1.13e-6**	**1.85e-6**
	L-face	1.07e-2	1.97e-3	3.01e-5	1.57e-5	R-face	2.37e-3	5.59e-4	3.31e-6	3.61e-5
VIP+	R-face	**8.07e-3**	**2.04e-4**	1.27e-6	**3.57e-7**	L-face	**6.90e-3**	**3.92e-4**	**2.51e-6**	**1.52e-5**
	L-face	3.37e-2	4.48e-4	**9.39e-7**	6.08e-7	R-face	1.54e-2	1.04e-3	8.23e-5	1.70e-3
V6A	R-face	**4.83e-3**	4.00e-3	**4.13e-6**	**1.13e-7**	L-face	**8.13e-3**	**1.37e-4**	**8.01e-7**	**6.57e-7**
	L-face	2.63e-2	**3.52e-3**	1.60e-5	1.11e-6	R-face	2.04e-2	2.18e-2	3.02e-4	1.99e-4
LIP+	R-face	**4.37e-3**	**4.46e-5**	**4.98e-6**	**2.65e-6**	L-face	3.97e-2	**1.22e-3**	**9.47e-4**	**6.13e-4**
	L-face	6.09e-2	6.25e-4	2.68e-4	8.94e-6	R-face	**6.70e-3**	3.21e-2	4.45e-3	2.47e-2
FEF	R-face	**1.53e-2**	**3.44e-4**	**1.11e-6**	3.78e-7	L-face	**1.13e-2**	**1.57e-3**	6.75e-5	**1.98e-5**
	L-face	4.04e-2	1.85e-3	5.45e-6	**2.81e-8**	R-face	2.78e-2	2.05e-2	**6.17e-5**	2.87e-3
DLPFC	R-face	**2.47e-2**	**2.67e-2**	**9.74e-4**	1.51e-2	L-face	**2.69e-3**	**1.42e-3**	1.55e-3	**8.32e-5**
	L-face	7.94e-2	9.12e-2	7.45e-2	**7.51e-3**	R-face	8.98e-3	8.04e-3	**9.42e-4**	1.97e-3
7b	R-face	**1.80e-3**	3.26e-2	**2.83e-6**	**1.88e-6**	L-face	**7.46e-4**	**3.53e-2**	**5.13e-5**	**4.94e-6**
	L-face	2.82e-3	**2.81e-2**	7.46e-6	4.32e-5	R-face	3.61e-2	1.59e-1	1.44e-3	4.98e-4
PV/S2	R-face	**5.18e-4**	7.81e-2	**2.32e-7**	**6.78e-8**	L-face	**9.25e-4**	**2.65e-1**	**2.91e-4**	**1.91e-5**
	L-face	2.49e-3	**5.60e-2**	1.00e-6	1.20e-7	R-face	6.30e-2	6.93e-1	6.29e-3	5.28e-3
AIC	R-face	1.80e-1	5.02e-1	**9.37e-5**	1.01e-4	L-face	**4.29e-2**	**8.30e-2**	**1.24e-2**	**5.16e-4**
	L-face	**3.47e-2**	**7.33e-3**	2.71e-3	**1.12e-5**	R-face	3.76e-1	5.72e-1	2.28e-2	9.30e-3
aPCu	R-face	**1.24e-2**	**2.59e-2**	**1.03e-3**	**3.26e-5**	L-face	**1.71e-2**	**2.17e-3**	**2.78e-5**	**3.88e-6**
	L-face	4.31e-2	4.51e-2	2.56e-3	2.64e-4	R-face	6.80e-2	1.18e-1	2.32e-3	2.14e-3
SMA	R-face	4.18e-2	3.99e-2	**1.60e-7**	2.03e-7	L-face	**6.38e-3**	**7.88e-3**	1.14e-5	**5.97e-7**
	L-face	**8.90e-3**	**3.84e-3**	4.08e-6	**3.16e-8**	R-face	2.96e-2	3.68e-2	**1.51e-6**	1.32e-5
CaS-p	R-face	**2.46e-2**	1.95e-1	3.98e-4	4.36e-5	L-face	**2.78e-2**	**6.42e-2**	**4.43e-5**	6.17e-5
	L-face	4.12e-2	**5.22e-2**	**1.60e-4**	**1.98e-5**	R-face	4.32e-2	2.10e-1	3.21e-4	**3.36e-5**
MI/SI	R-face	**3.57e-1**	9.97e-1	**5.98e-8**	6.87e-8					
	L-face	6.21e-1	**9.02e-1**	7.98e-7	**4.47e-9**					
SI	R-face	**2.03e-1**	**1.98e-1**	1.67e-6	4.42e-7					
	L-face	1	3.54e-1	**9.66e-7**	**1.75e-7**					
Subc	R-face	**3.34e-2**	**1.38e-4**	2.70e-1	1.48e-2	L-face	6.44e-2	3.45e-3	**8.19e-2**	**2.43e-2**
	L-face	5.69e-2	3.57e-3	**5.57e-2**	**1.12e-2**	R-face	**4.40e-2**	**1.97e-4**	2.80e-1	3.03e-2
Pulvinar	R-face	6.09e-2	**4.56e-2**	9.52e-4	2.23e-5	L-face	**8.64e-2**	**6.73e-2**	**2.71e-3**	**2.43e-4**
	L-face	**2.14e-2**	9.34e-2	**8.00e-4**	**1.85e-5**	R-face	1.28e-1	8.48e-2	1.15e-2	5.49e-4
V3B	R-face	**7.29e-3**	**2.37e-5**	**1.08e-5**	**2.21e-5**	L-face	3.21e-1	**2.66e-3**	**1.39e-3**	**9.28e-4**
	L-face	2.17e-1	5.22e-3	4.68e-3	7.84e-4	R-face	**5.02e-2**	8.47e-2	9.30e-3	4.26e-2
V2v/V3v	R-face	**2.34e-1**	**1.64e-3**	**5.96e-5**	**2.34e-4**	L-face	**1.85e-1**	**6.11e-5**	**3.43e-6**	**7.74e-6**
	L-face	4.42e-1	1.26e-1	2.36e-2	4.41e-2	R-face	3.17e-1	5.50e-1	7.61e-3	3.39e-2
O.C.	R-face	**2.75e-2**	**6.19e-7**	**8.65e-10**	**2.90e-9**	L-face	**1.75e-2**	**1.89e-7**	**1.28e-10**	**3.24e-10**
	L-face	7.61e-2	8.77e-3	6.60e-5	8.56e-5	R-face	5.80e-2	1.80e-2	7.94e-5	6.72e-5

sROIs above/below the central divider: see Figs. 7/8. Each estimated *p*-value (Bonferroni corrected, n =11) is reported in E-notation format (e.g., 5.01e-2 denotes a value of 5.01×10^{-2} or 0.0501). Shaded/unshaded: Contralateral/ipsilateral stimuli; O.C.: occipital cluster; Bolded number: the lower of the two *p*-values in response to left-face and right-face stimuli under each event type.
